# Self-Assembled Silk Fibroin-Based Aggregates for Delivery of Camptothecin

**DOI:** 10.3390/polym13213804

**Published:** 2021-11-03

**Authors:** Javier Pérez Quiñones, Cornelia Roschger, Andreas Zierer, Carlos Peniche-Covas, Oliver Brüggemann

**Affiliations:** 1 Institute of Polymer Chemistry, Johannes Kepler University Linz, Altenberger Straße 69, 4040 Linz, Austria; oliver.brueggemann@jku.at; 2 Department for Cardiac-, Vascular- and Thoracic Surgery, Johannes Kepler University Linz, Kepler University Hospital GmBH, Altenberger Straße 69, 4040 Linz and Krankenhausstraße 7a, 4020 Linz, Austria; cornelia.roschger@jku.at (C.R.); or andreas.zierer@kepleruniklinikum.at (A.Z.); 3 Facultad de Química, Universidad de La Habana, Zapata S/N entre G y Carlitos Aguirre, La Habana 10400, Cuba; cpeniche2015@yahoo.com

**Keywords:** silk fibroin, camptothecin, controlled release, nanoaggregates, antitumor activity

## Abstract

A water-soluble hydrolysate of silk fibroin (SF) (~30 kDa) was esterified with tocopherol, ergocalciferol, and testosterone to form SF aggregates for the controlled delivery of the anticancer drug camptothecin (CPT). Elemental analysis and ^1^H NMR spectroscopy showed a degree of substitution (DS) on SF of 0.4 to 3.8 mol %. Yields of 58 to 71% on vitamins- and testosterone-grafted SF conjugates were achieved. CPT was efficiently incorporated into the lipophilic core of SF aggregates using a dialysis–precipitation method, achieving drug contents of 6.3–8.5 wt %. FTIR spectra and DSC thermograms showed that tocopherol- and testosterone-grafted SF conjugates predominantly adopted a β-sheet conformation. After the esterification of tyrosine residues on SF chains with the vitamin or testosterone, the hydrodynamic diameters almost doubled or tripled that of SF. The zeta potential values after esterification increased to about −30 mV, which favors the stability of aggregates in aqueous medium. Controlled and almost quantitative release of CPT was achieved after 6 days in PBS at 37 °C, with almost linear release during the first 8 h. MCF-7 cancer cells exhibited good uptake of CPT-loaded SF aggregates after 6 h, causing cell death and cell cycle arrest in the G2/M phase. Substantial uptake of the CPT-loaded aggregates into MCF-7 spheroids was shown after 3 days. Furthermore, all CPT-loaded SF aggregates demonstrated superior toxicity to MCF-7 spheroids compared with parent CPT. Blank SF aggregates induced no hemolysis at pH 6.2 and 7.4, while CPT-loaded SF aggregates provoked hemolysis at pH 6.2 but not at pH 7.4. In contrast, parent CPT caused hemolysis at both pH tested. Therefore, CPT-loaded SF aggregates are promising candidates for chemotherapy.

## 1. Introduction

Cancer is becoming the mayor cause of death worldwide in the present century. In 2018, an estimate of 18.1 million new cancer cases were detected and 9.6 million cancer patients died [1,2]. Lung cancer is the most diagnosed and lethal cancer with 11.6% and 18.4% of the total cases and cancer-related mortality. It is followed by breast and prostate cancer as well as colorectal cancer in terms of incidence and mortality [1]. Conventional chemotherapy is still widely used in medical treatment of most cancers and tumors usually in combination with surgery, radiotherapy, and novel nanomedicines. These include genetically designed and personalized drugs, prodrugs, multiresponsive drug delivery systems, and thermal phototherapy, among others [2,3,4]. The anticancer drugs irinotecan and topotecan, two CPT derivatives, are used to treat lung cancer, colon cancer, metastatic or resistant breast cancer, and ovarian cancer. A liposome formulation of irinotecan (Onivyde) was newly accepted by the FDA and EU control authorities for the management of metastatic pancreatic cancer [5,6,7,8]. CPTs are the only marketed topoisomerase I inhibitors. However, CPTs provoke severe side effects and CPT lactones have a short serum half-life [9,10]. This motivates intense research on the preparation of different analogues and novel drug delivery systems [11,12]. However, few CPT delivery systems are commercially available and approved for antitumor therapy in addition to Onivyde [13]. In search of new nanomedicines also capable of tumor-targeted drug delivery [14], we worked on CPT encapsulation in tocopherol-, ergocalciferol-, and testosterone-modified hyaluronic acid and cellulose nanogels (2–13 wt % of CPT), with sustained drug release and good cytotoxic activity on MCF-7 cancer cells [15,16]. It was found that testosterone-polysaccharide and vitamin-polysaccharide conjugates exhibited almost negligible release of testosterone and vitamins, with slow CPT release during 8 h in PBS at pH 7.4 and 37 °C [15,16]. On the other hand, significant co-delivery of encapsulated CPT and covalently linked testosterone and vitamins from the nanogels was observed when the release experiments were performed in slightly acid conditions (PBS, pH 6.0, 37 °C), simulating the cancer tissue environment [15]. Tocopherol (vitamin E), ergocalciferol (vitamin D2), and testosterone were chosen as biocompatible modifiers of hydrophilic polymers for anticancer drug delivery because of their antioxidant, cardiovascular-protective, and anticancer effects [17,18]. Particularly, testosterone used in combination with tamoxifen and anastrozole in breast cancer treatments has shown a reduced cancer relapse and better prognosis [18]. Similarly, vitamin D2 intake is associated with reduced incidence and mortality of breast, colorectal, kidney, and lung cancers [17]. These findings encouraged us to synthesize tocopherol-, ergocalciferol- and testosterone-grafted nanocarriers based on biocompatible silk fibroin (SF) for the hydrophobic encapsulation of CPT and later controlled delivery, with anticipated good cytotoxicity against cancer cells and decreased side effects. The rational of using SF as a polymer carrier of CPT was the exhaustive use of SF materials in diverse biomedical applications [19,20,21,22,23] as well as straightforward SF functionalization via tyrosine esterification [24,25]. In this sense, we have efficiently synthesized steroids-grafted SF derivatives via carbodiimide-mediated esterification for agrochemicals applications [25]. Furthermore, SF hydrolysate has shown slight antiproliferative effect on MCF-7, A549, and H460 cancer cells [26].

SF is obtained from the *Bombyx mori* cocoons. It is formed by a long 350–370 kDa protein and two short ones of 25–27 kDa each [20]. SF proteins are composed of 18 amino acids. Its major components are alanine, glycine, tyrosine, and serine [27]. On the other hand, biocompatible SF hydrolysates are sometimes preferred over natural SF for medical use due to definite chemical composition, purity, and narrow molecular sizes distribution [28].

Hereby, a SF fraction (~30 kDa) was functionalized with tocopherol, ergocalciferol or testosterone to form SF aggregates, which efficiently encapsulated and delivered CPT. The chemical structure, thermal, and aggregate properties were studied. In addition, the CPT-controlled release behavior and cytotoxicity of blank and CPT-loaded SF aggregates were assessed. The high CPT content found in CPT-loaded testosterone-grafted SF aggregates in spite of the significantly lowest degree of substitution of SF with testosterone must be noted. It is also worth mentioning the significant shrinkage of SF aggregates in water once CPT was encapsulated in the hydrophobic core. The antiproliferative effect of CPT on MCF-7 cells remained unaltered after CPT loading in the SF aggregates. Good uptake of CPT-loaded SF aggregates was observed on MCF-7 cancer cells after 6 h. MCF-7 3D spheroids also showed good uptake of the CPT-loaded nanocarriers, with significant lower cell viability than when parent CPT is evaluated. As far as we know, the preparation of CPT-loaded SF carriers for controlled delivery and anticancer applications has not been attempted before.

## 2. Materials and Methods

### 2.1. Materials

SF hydrolysate (98% purity, ~30 kDa) (Leap Labchem Co., Hangzhou, China) was purified using 12–14 kDa cellulose dialysis membranes against water (2 L) at 4 °C for 48 h. During this time, water was replaced 4 times. After that, an SF fraction was obtained as white soft flakes. (*S*)-(+)-Camptothecin (CPT) was purchased from Alfa Aesar (Alfa Aesar GmbH & Co KG, Karlsruhe, Germany). Testosterone hemisuccinate, tocopherol hemisuccinate, and ergocalciferol hemisuccinate, as well as their *N*-hydroxysuccinimide esters, were previously synthesized [29,30].

### 2.2. Preparation of Ergocalciferol-, Tocopherol-, and Testosterone-Grafted SF Aggregates

Ergocalciferol-, tocopherol-, and testosterone-grafted SF were prepared via the esterification of tyrosine fragments of SF with testosterone and vitamin hemisuccinate *N*-hydroxysuccinimide esters to prevent undesired cross-linking of SF as previously reported [25]. Ergocalciferol-grafted SF (SF1) was obtained as a brown powder. Tocopherol-grafted-SF (SF2) and testosterone-grafted SF (SF3) appeared as white powders after lyophilization (characterization data of SF1–SF3 is included in the Supplementary Material). These SF conjugates formed aggregates of particles in aqueous medium when stirred overnight at 1 mg/mL in water or PBS.

### 2.3. CPT Loading in SF Aggregates

Hydrophobic CPT was incorporated in the lipophilic core of ergocalciferol-, tocopherol-, and testosterone-grafted SF aggregates using a dialysis-precipitation method with lyophilization [4,16]. To this end, 10 mg of SF1, SF2, or SF3 and 1 mg of CPT in DMSO were stirred overnight at 25 °C in darkness. The formation of CPT-loaded SF aggregates and removal of excess CPT was carried out by dialysis against distilled water (2 L) for 5 h with one replacement. CPT–SF1 (slightly brown), CPT–SF2 and CPT–SF3 (white powders) were obtained after lyophilization.

### 2.4. CPT Content and Sustained Delivery

The content of CPT in CPT-loaded SF aggregates and related parameters (loading efficiency and yield of aggregates), as well as *in vitro* released CPT during drug release studies were determined by UV spectrophotometry (PerkinElmer Ltd., Buckinghamshire, UK), based on calibration curves of CPT in DMSO and PBS (pH 7.4) (Figures S1–S3) (CPT $\varepsilon_{368}^{DMSO}$= 21,006 M^−1^ cm^−1^, $\varepsilon_{370}^{PBS}$= 26,930 M^−1^ cm^−1^).

CPT delivery studies were conducted at 37 °C in PBS (pH 7.4). First, 5 mg of CPT–SF1, CPT–SF2 or CPT–SF3 in 2 mL of PBS were dialyzed against 10 mL of PBS. The 10 mL PBS was replaced with fresh media, and CPT delivered was determined using UV spectroscopy (CPT λ_emission_ = 370 nm).

### 2.5. Characterization

Molecular weight determinations of SF were performed with a Viscotek GPCmax gel permeation chromatograph (GPC) provided with a PFG column from PSS (Mainz, Germany) (300 mm × 8 mm and 5 µm particle size) and a Viscotek TDA 305 Triple Detector Array (Malvern, Kassel, Germany) [25]. Calibration was performed by triple detection (refractive index, right angle light scattering and viscometer) with PSS polystyrene standards [25]. The purified SF sample was eluted with 10 mM LiBr in DMF and a flow rate of 0.75 mL/min at 60 °C.

ATR-FTIR spectra of SF conjugates were obtained using a Perkin Elmer Spectrum 100 FT-IR spectrophotometer (PerkinElmer Ltd., Buckinghamshire, UK) with an ATR accessory and a resolution of 4 cm^−1^ [25].

Differential scanning calorimetry (DSC) profiles of the samples were obtained using a TA Instruments DSC Q2000 (TA Instruments, Eschborn, Germany) with around 5 mg samples. A 10 °C/min ramp and 20 mL/min nitrogen flow were used for all runs. Samples were cooled and heated from 0 to 300 °C in sealed aluminum pans with holes. Thermogravimetric analyses (TGA) were carried out on a TGA Q5000 instrument (TA Instruments, Eschborn, Germany). First, 5 mg of samples on platinum pans were heated from 40 to 800 °C. A 10 °C/min ramp and 25 mL/min of nitrogen flow were used for the measurements.

^1^H NMR spectra were registered at 25 °C in solution (DMSO-d_6_, δ = 2.51 ppm) [31] in a Bruker Avance III 300 spectrometer (Bruker, Faellanden, Switzerland) at 300 MHz frequency.

UV-Vis spectra were recorded on a Perkin Elmer Lambda 25 UV/VIS spectrophotometer (PerkinElmer Ltd., Buckinghamshire, UK). Dynamic light scattering (DLS) determinations were carried out on a Malvern Zetasizer Nano ZS (Malvern Instruments Ltd., Malvern, UK) operated at 173°. Measurements were made in triplicate at room temperature. The SF aggregates in water or PBS at 0.5 mg/mL were filtered through a 0.45 µm nylon filter and measured in a DTS1070 disposable cuvette. Transmission Electron Microscopy (TEM) images were registered using a Jeol JEM-2011 FasTEM (Jeol Ltd., Tokyo, Japan) at 100 kV [25]. SF solutions were placed on Pioloform–coated copper grids, using negative staining with 1% uranyl acetate for contrast enhancement. TEM images were processed, and the size of aggregates and particles were measured using Gatan Digital Micrograph 3.10.1 (Pleasanton, CA, USA).

Atomic Force Microscopy (AFM) images (2 µm × 2 µm) were taken with an MFP 3D-Stand Alone AFM (Asylum Research, Oxford, UK) with the Olympus cantilever OMCL-AC160TSA (Olympus Europa SE & Co. KG, Hamburg, Germany) at a resonant frequency of 300 kHz and spring constant of 26 N/m, 50–70% set point, and scan rate of 1 Hz [25]. A 70 µL droplet of 1 mg/mL water solution of SF conjugates was placed on a silicon wafer and spin coated at 40 Hz for 6 s. AFM images were processed and statistical parameters of particles were obtained using Gwyddion 2.45 (Brno, Czech Republic).

### 2.6. Viability Assay

To determine the effect of the nanoparticles on the viability of the human breast adenocarcinoma cell line MCF-7, 2,3-bis(2-methoxy-4-nitro-5-sulfophenyl)-2*H*-tetrazolium-5-carboxanilide assay (XTT) (Sigma-Aldrich, St. Louis, MO, USA) was used. Cells were grown in Dulbecco’s modified Eagle’s medium (DMEM), with 10% fetal bovine serum, 1% penicillin–streptomycin, and 1% L-glutamine. The MCF-7 cell line was a gift from Prof. Dr. Barbara Krammer, Paris-Lodron-University, Salzburg, Austria. Cell suspensions were cultivated in 96-well tissue culture plates in triplicate (4 × 10^4^ cells/well). The day after, cells were exposed to dispersions of SF aggregates loaded or not with CPT at various concentrations in serum-free medium. After an additional incubation period of 48 h, the medium was discarded, and the cells were supplemented with complete DMEM with 50 μL of XTT. After 3 h at 37 °C in the dark, the absorbance at 490 nm was measured with a GloMax^®^ Multimode Microplate Reader (Promega, Madison, WI, USA). Three independent experiments were performed. Samples were normalized to untreated controls. Data were analyzed using GraphPad Prism 6.0 (San Diego, CA, USA).

### 2.7. Cell Uptake

For cell uptake analysis, 3 × 10^4^ cells in complete growth medium were seeded into a 8-well µ-slide (ibidiTreat, Ibidi, Graefeling, Germany). The next day, medium was aspirated, and CPT-loaded SF aggregate dispersions at 0.1 mg/mL in serum-free medium were added. After 4 h of incubation at 37 °C in the dark, cells were exposed to 50 nM LysoTracker Yellow HCK-123 (Invitrogen, Waltham, MA, USA) for an additional 2 h. Subsequently, cells were imaged using an Olympus IX73 inverted microscope. For the CPT-loaded SF aggregates, the DAPI channel (λ_excitation_ = 345 nm and λ_emission_ = 455 nm) was used, and for the LysoTracker Yellow HCK-123, the FITC channel (λ_excitation_ = 494 nm and λ_emission_ = 518 nm) was used.

### 2.8. Annexin V/PI Assay

MCF-7 cells (1 × 10^5^ cells/well) were plated overnight in 12-well culture plates. Subsequently, the medium was removed and serum-free medium was added, containing the compounds at various concentrations. After 48 h, supernatants were transferred to Eppendorf tubes. Cells were harvested with 500 μL of Accutase^®^ solution (Sigma-Aldrich, St. Louis, MO, USA) and mixed with the supernatant. The suspension was centrifuged at 1500 rpm for 5 min and washed twice with 1 mL of DPBS. The obtained pellet was stained for 15 min in 98.5 µL of 1X Annexin V Buffer with 1 µL of Annexin V-APC (Immuno-Tools, Friesoythe, Germany) and 0.5 µL of 1 mg/mL propidium iodide (PI, Sigma-Aldrich, St. Louis, MO, USA). Subsequently, 200 µL of 1X Annexin V Buffer were added and measured with the CytoFLEX flow cytometer (Beckman Coulter, Brea, CA, USA). At least three different experiments were conducted and analyzed with the Kaluza 1.5a software (Beckman Coulter, Brea, CA, USA).

### 2.9. Cell Cycle Analysis

First, 5 × 10^5^ cells/well in 6-well plates grown overnight were treated with nanoparticles at different concentrations for 72 h. Afterwards, the supernatants were transferred to Eppendorf tubes, and cells were detached with 500 μL Accutase^®^ solution. After centrifugation (5 min at 1500 rpm), the cell pellet was washed with 1 mL of DPBS. The supernatant was discarded, and the pellet was re-suspended in 100 μL DPBS. To fix the cells, 1 mL of ice-cold 70% ethanol was added. Fixed cells were frozen for about 1 h at −20 °C. After washing, cells were stained with 5 μL staining solution (0.4 mg/mL PI, 5 μL of 1 mg/mL RNase solution in DPBS) for 15 min at 37 °C in the dark. Subsequently, cells were measured with the CytoFLEX flow cytometer and analyzed with the Kaluza 1.5a software (both Beckman Coulter, Brea, CA, USA).

### 2.10. Spheroid Generation

First, 1 × 10^4^ cells were seeded in a BIOFLOAT FLEX (faCellitate, Mannheim, Germany) coated non-TC treated U-bottom 96-well plate. Subsequently, the plate was centrifuged for 5 min at 300× *g*, to facilitate cell aggregation and subsequent spheroid formation.

### 2.11. Spheroid Uptake

After an incubation period of 4 days, the spheroids were treated with 0.1 mg/mL of the indicated compounds. After 72 h, the spheroids were washed and stained with PI for 10 min to visualize dead cells. Cells were counterstained using Hoechst 33342 (Fluka, Buchs, Switzerland). Fluorescence imaging was performed with an Olympus IX73 inverted microscope provided with DAPI channel for Hoechst 33342 and the CPT-loaded SF aggregates (λ_excitation_ = 345 nm and λ_emission_ = 455 nm) and Cy3 channel for PI (λ_excitation_ = 550 nm and λ _emission_ = 565 nm).

### 2.12. Cell Viability Analysis of Spheroids

After an incubation period of 4 days, the spheroids were treated with increasing concentrations of the compounds in serum-free medium. To measure the cell viability with the CellTiter-*Glo*^®^ *3D* Cell Viability Assay (Promega, Madison, WI, USA), the spheroids were deposited into a 96-well plate after 72 h and processed as described in manufacturer’s protocol. The Luminescence was measured with the GloMax (Promega, Madison, WI, USA).

### 2.13. Hemolysis Assay

First, 4 mL of whole blood were obtained from a healthy human donor, which were directly drawn into Vacuette^®^ EDTA tubes (Greiner-Bio-One, Kremsmuenster, Austria) to prevent coagulation. The blood was centrifuged for 5 min at 2500× *g* to obtain a red blood cell (RBC) pellet, the plasma was discarded and the RBCs were washed twice with 150 mM NaCl (Merck, Darmstadt, Germany) and in triplicate with PBS at pH 7.4. Consequently, the RBCs were diluted 1:50 with PBS at pH 6.2 or pH 7.4 and 200 μL RBC suspension were incubated with the nanoparticles at 37 °C for 24 h, without CO_2_. Samples incubated with 1% Triton-X (Merck, Darmstadt, Germany) or PBS pH 7.4 instead of nanoparticles served as positive and negative control, respectively. Samples were centrifuged at 500× *g* for 5 min, and 100 μL of the supernatant was placed into a flat-bottom 96-well plate. Absorbance was determined with a GloMax microplate reader (Promega, Madison, WI, USA) at 405 nm. The percent hemolysis was determined as follows:

$$\%\;\mathrm{hemolysis}\;=\left[\frac{\left(\mathrm{Absorbance}\;\mathrm{sample}\;-\mathrm{Absorbance}\;\mathrm{negative}\;\mathrm{control}\right)}{\mathrm{Absorbance}\;\mathrm{positive}\;\mathrm{control}}\right]\times 100$$

### 2.14. Statistical Analyses

All data are normalized and reported as average value ± standard deviation. Measurements were evaluated using a one-way ANOVA with Tukey’s post-test for multiple comparison analysis (Statgraphics Plus 5.1). Statistically significant sample means were defined with a significance threshold set at 5% (*p* < 0.05), where * or no marker at all is used for means with significant differences (*p* < 0.05), and ns is used for means with no significant differences (*p* > 0.05).

## 3. Results

### 3.1. Synthesis and Characterization

**Figure 1:**
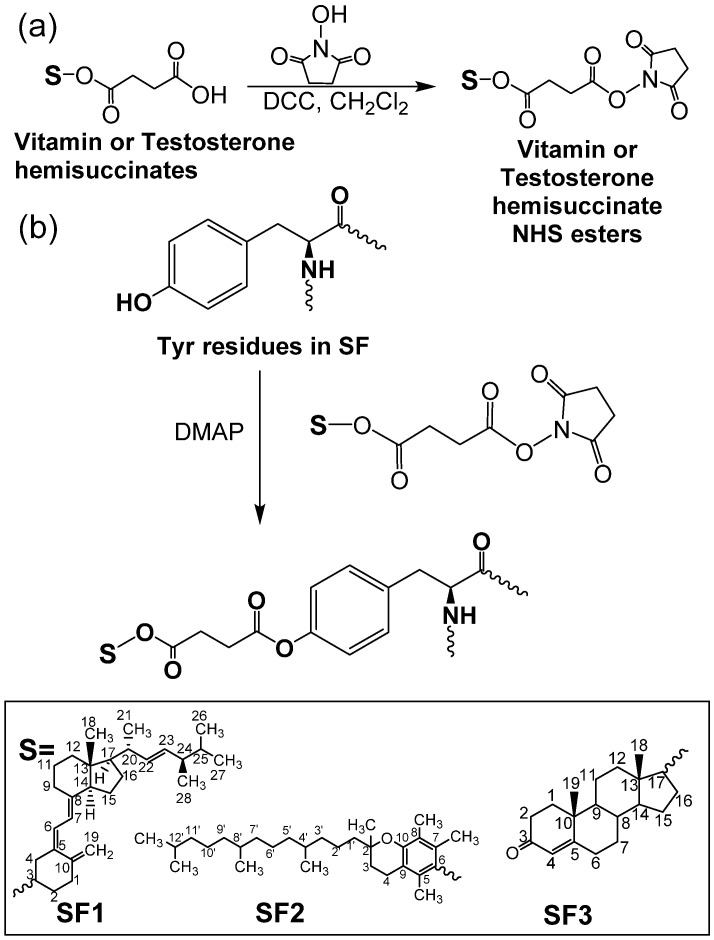
Synthetic route to vitamins–SF and testosterone–SF. (**a**) Formation of *N*-hydroxysuccinimide derivatives of vitamin and testosterone hemisuccinate; (**b**) reaction of tyrosine moieties in SF with vitamin and testosterone *N*-hydroxysuccinimide derivatives.

Figure 1 illustrates the structures of vitamin- and testosterone-grafted SF conjugates synthesized via the ester formation of tyrosine moieties in SF with vitamins and testosterone. Tyrosine residues of SF were chosen as the target point for the synthesis of SF conjugates because of the higher nucleophilicity of its –OH groups and the high tyrosine content in SF (approximately 5 mol %) [27,32]. Approximately 70–80% of tyrosine residues in SF were esterified with the vitamin hemisuccinates, while testosterone hemisuccinate reacted with about 9% of the tyrosine fragments (Table 1). Testosterone hemisuccinate was much less reactive than vitamin hemisuccinates in the esterification of SF, as previously observed in the esterification of cellulose ethers [16]. DS values estimated from ^1^H NMR for SF1 and SF2 were similar to the values calculated from elemental analyses. The effect of the hydrophobic functionalization of SF on the SF structure, morphology, and properties was thoroughly studied.

### 3.2. Structural Characterization and Morphological Studies

**Figure 2:**
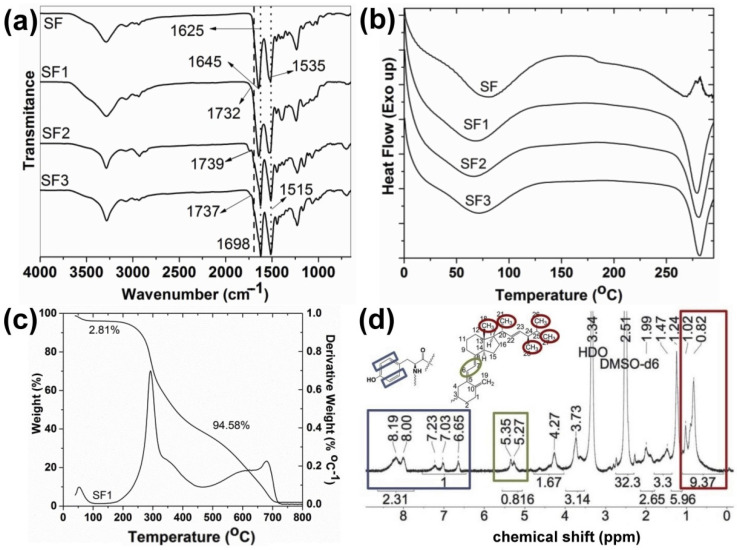
(**a**) IR spectra of native and modified SF; (**b**) DSC curves of SF and SF1–SF3 conjugates; (**c**) TGA thermogram of SF1 with weight loss indicated; (**d**) ^1^H NMR spectrum of SF1 in DMSO-d_6_ (see structures in Figure 1).

IR spectroscopy confirmed the functionalization of SF with vitamins and testosterone hemisuccinates. C=O peaks of ester bonds and free carboxylic acid groups in vitamins and testosterone hemisuccinates are observed between 1728–1746 cm^−1^ and 1695–1702 cm^−1^, respectively (Figure S4) [16,33,34]. Once the tyrosine residues in SF were esterified with vitamins and testosterone hemisuccinates, C=O peaks of the vitamin- and testosterone-grafted SF ester bonds were observed at 1732–1739 cm^−1^ (Figure 2a, characterization data in Supplementary Material) [16,25]. SF with intense peaks observed at 1535 and 1645 cm^−1^ (silk I) must adopt random coil and β-turns structures [19]. Synthesized SF1–SF3 must consist of a β-sheet structure, as evidenced with intense IR peaks at 1515 and 1625 cm^−1^ (silk II) [19]. The effect of vitamins and testosterone on the thermal behavior and related properties of SF was also studied (Figure 2b,c). SF1–SF3 exhibited an endothermic peak at 66–71 °C with a peak enthalpy (ΔH) of 149–213 J/g and related weight reduction of 2.4–4.2% (Figure 2b,c, and Figures S5 and S6). This peak might be ascribed to the loss of SF-bound water [35]. Parent SF showed an endothermic peak at 80 °C with a related enthalpy of 209.4 J/g and 7.9% weight loss (Figure 2b and Figure S7) [25]. The glass transition of native SF was detected at 182 °C, which is near the 178 °C for amorphous SF [35,36]. This glass transition characteristic of SF (silk I) is associated to changes in the β-turns and random coils [35]. It was not detected in the SF1–SF3 conjugates. Partial pyrolysis of SF was detected in native and modified SF as endothermic peaks at 266–280 °C, with ΔH of 43 J/g and 100–133 J/g (Figure 2b). The total pyrolysis of raw and modified SF provoked a major weight loss of 91–96% (Figure 2c and Figures S5–S7).

^1^H NMR spectroscopy of vitamin- and testosterone-grafted SF in solution also corroborated the functionalization with vitamins and testosterone hemisuccinates of SF tyrosine amino acids (Figure 2d). The characteristic tyrosine protons appearing at 6.7–8.2 ppm (Figure S8) were taken as a reference to calculate the DS of SF1 and SF2, with the aid of the distinctive vitamin signals at 0.82–1.02 ppm (CH_3_– groups H18, H21, H26, H27, and H28 in ergocalciferol; 4′CH_3_–, 8′CH_3_–, and 12′CH_3_– in tocopherol), and at 5.27–5.35 ppm (=CH–CH= group H6 and H7 in ergocalciferol) (Figure 2d and Figure S9) [16]. The proton NMR spectra of SF3 also showed the characteristic testosterone peaks at 0.84–0.86 ppm (CH_3_– groups H18 and H19 in testosterone) and at 5.34 ppm (O=CC–CH= group H4 in testosterone) (Figure S10). However, the low signal to noise ratio in the ^1^H NMR spectra of SF3 resulted in an underestimated integration of tyrosine aromatic protons, and a proper DS estimation was not suitable.

DLS studies revealed the effect of SF esterification with vitamins and testosterone on the hydrodynamic size of SF aggregates and their zeta potential in water. Table 2 shows that the esterification of SF with the vitamins or testosterone provoked a significant change of their hydrodynamic parameters. Almost doubled or tripled hydrodynamic diameters and doubled zeta potential values were observed after the esterification of tyrosine residues on SF chains.

SF1–SF3 aggregates presented hydrodynamic sizes and zeta potential values in water of 554 to 653 nm and −28.3 to −32.2 mV, respectively. It seems that the intermolecular hydrophobic interaction of vitamins or testosterone groups grafted on SF chains caused stabilization of the SF aggregates in aqueous medium, with zeta potentials around −30 mV [37]. In general, the hydrodynamic sizes increased in PBS for native SF and SF aggregates when compared to the same aggregates dispersed in distilled water, except for SF1 aggregates. On the other hand, the hydrophobic loading of CPT in the inner core of SF aggregates resulted in a significant hydrodynamic sizes reduction of 11% to 59%, which was probably due to the stronger hydrophobic interactions of CPT molecules with vitamins and testosterone grafted on SF chains.

The strong hydrophobic interactions between CPT and lipophilic vitamins and testosterone allowed achieving a high CPT content in the prepared CPT–SF1 to CPT–SF3 aggregates, with encapsulation efficiencies ($EE\;\%=\left(\frac{weight\;of\;CPT\;loaded\;in\;particles}{weight\;of\;feeding\;CPT}\right)\times 100)$ ranging from 60% to 84% (Table 2). Particularly interesting was the high CPT content found in CPT–SF3 aggregates and the associated high encapsulation efficiency achieved (67%) in spite the low testosterone content related to a DS of 0.4 mol % in SF3 conjugates. However, CPT–SF3 aggregates exhibited the smaller hydrodynamic diameters in aqueous medium. This might be due to the additional π−π interactions between the phenyl groups of tyrosine residues in SF with the aromatic backbone of CPT.

**Figure 3:**
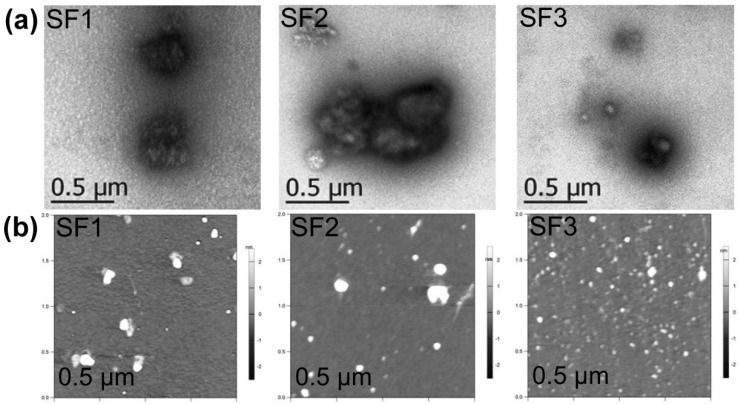
(**a**) TEM micrographs of SF1–SF3 dried aggregates at 21,000× magnification; (**b**) AFM micrographs of SF1–SF3 dried aggregates (see structures in Figure 1).

SF1–SF3 dried aggregates appeared on TEM as irregular grain aggregates of around 50–800 nm (Figure 3a), with most of the small SF particles observed as agglomerates with undefined borders (Figure S11). AFM showed SF nanoaggregates with 45 to 75 nm sizes for SF1–SF3 (Table 2, Figure 3b, Table S1 and additional AFM images in Supplementary Material). It must be noted that the size and distribution of dried SF aggregates is better calculated from AFM measurements due to a proper statistical analysis of SF samples (a representative number of SF particles is present in the 2 µm × 2 µm AFM images)**.** Furthermore, the spin-drying process of AFM samples ensures a better dispersion of SF aggregates before measurements.

### 3.3. Drug Release

**Figure 4:**
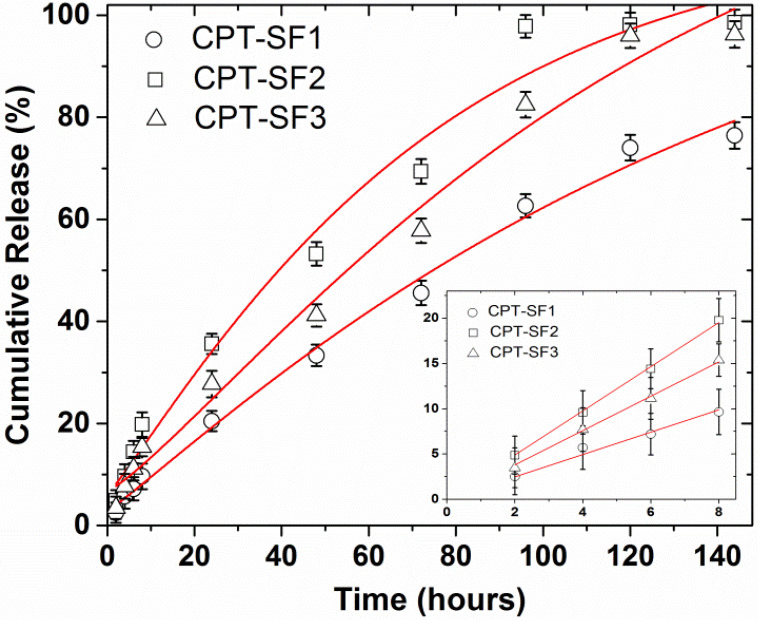
CPT delivery of CPT-loaded SF aggregates in PBS, 37 °C, adjusted to a SWeibull2 function; CPT releases for initial 8 h, adjusted to a linear function (inset) (see structures in Figure 1). Mean ± standard deviation (*n* = 3).

CPT delivery of CPT-loaded SF aggregates in PBS (pH 7.4) at 37 °C is presented in Figure 4. All CPT releases appeared linear with a slope 1.23 to 2.44%/h during the initial 8 h (Figure 4 and Table S2). The almost linear CPT release rate profiles observed during the initial 8 h can be due to a frequent dialysate replacement with fresh PBS. A frequent replacement of dialysate that contains the poorly soluble CPT with fresh release medium must affect the diffusion rate of CPT from the core of the SF aggregates and through the dialysis membrane. CPT was almost quantitatively released after 6 days. The almost quantitative CPT release after 6 days in PBS is due to the repeated replacement of the dialysate in every time point studied, which forced the diffusion of almost all CPT encapsulated in the SF aggregates after 10 replacements of the medium release (10 mL of PBS). CPT–SF2 and CPT–SF3 achieved a CPT release of approximately 97%, while the bigger CPT–SF1 aggregates carrying more CPT released 76% of the encapsulated anticancer drug. Furthermore, all the CPT release profiles adjusted well to the Weibull model that describes the drug release from a matrix [38,39]. To this end, kinetics data were fitted following a SWeibull2 distribution (Cumulative Release (%) = a − (a − b)*exp(−(k*Time(hours))^d^), with adjusted R^2^ ranging from 0.9760 to 0.9918 and d values of 1.1 and 1.2 associated to a complex drug release mechanism (Figure 4 and Table S3) [39]. The CPT release kinetic data were also adjusted to the Korsmeyer–Peppas model (log(Cumulative Release (%)) *=* k*log(Time(hours) + m) to get an insight into the molecular interactions and factors dominant on the CPT release behavior (Table S4 and Figure S12) [16,38,40]. The slope of the linear fitting ranged from 0.72 to 0.78, which is related to a non-Fickian drug release mechanism (anomalous diffusion mechanism) [40]. Further evaluation of the interaction between the anticancer drug with the hydrophobically modified SF matrix was carried out according to the changes of the IR peak of CPT at 1737 cm^−1^ in the CPT-loaded SF aggregates (Table S4 and Figure S13). To this end, the intensity of IR adsorption peaks at 1732 cm^−1^ and 1739 cm^−1^ in blank and CPT-loaded SF aggregates were normalized to the characteristic 1515 cm^−1^ adsorption peak in all spectra. The IR adsorption peaks at 1732 cm^−1^ and 1739 cm^−1^ of CPT-loaded SF aggregates exhibited a 0.4 to 1.6 increase in intensity when compared to blank SF1–SF3 aggregates (Figure S13). It might be due to the overlapping of the C=O adsorption peak at 1732 cm^−1^ and 1739 cm^−1^ of blank SF1 and SF2, or SF3 aggregates respectively, with the intense CPT adsorption in the CPT-loaded aggregates. A significant shift of the CPT adsorption peak from 1737 cm^−1^ in parent CPT to 1732 cm^−1^ in CPT–SF1 aggregates was observed. It is probably due to slightly stronger interactions between CPT and the SF1 polymer matrix. On the other hand, CPT–SF2 and CPT–SF3 showed a small shifting of CPT peak to 1739 cm^−1^. The key factor that determines the CPT release rate and total CPT delivered after 6 days (drug delivery capability) seems to be the structure of the synthesized SF aggregates, which affects the CPT–polymer matrix interactions. SF1 aggregates with β-turns, random coils, and β-sheet conformers (silk I and silk II structure) are able to better accommodate the hydrophobic CPT in the lipophilic core, which facilitates the CPT–polymer matrix interactions when compared to the SF2 and SF3 aggregates (silk II structure), with a predominant content of β-sheet conformers and β-sheet nanocrystals. That is why the SF1 aggregates showed more CPT encapsulated and slower CPT release rate when compared to the SF2 and SF3 aggregates.

### 3.4. Cytotoxic Activity and Cell Uptake

**Figure 5:**
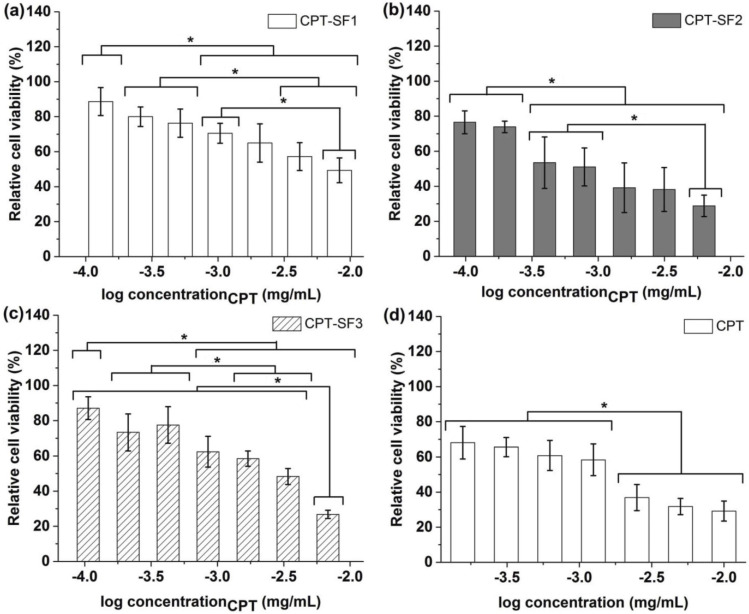
Relative viability of MCF-7 cells with (**a**) CPT-SF1 aggregates; (**b**) CPT-SF2 aggregates; (**c**) CPT-SF3 aggregates and (**d**) parent CPT (see structures in Figure 1). Mean ± standard deviation (*n* = 3). * represent means with significant differences (*p* < 0.05).

Figure 5 displays the viability of MCF-7 cells (normalized to untreated control) once treated with CPT-loaded SF aggregates or parent CPT. Non-loaded SF was not toxic to MCF-7 cells at 0.2 mg/mL, with a viability of (99 ± 5)% (Figure S14). Similarly, SF1–SF3 aggregates were not cytotoxic to MCF-7 (89–94% relative cell viability) at 0.1 mg/mL (Figure S14).

CPT hydrophobically loaded in SF aggregates provoked a significant cytotoxic effect, which was not observed with blank SF aggregates. Particularly, CPT–SF2 with a CPT concentration between 10^−2.8^ and 10^−2.2^ mg/mL exhibited similar to slightly better cytotoxic activity than parent CPT evaluated at 10^−2.6^ to 10^−2^ mg/mL. However, all CPT-loaded SF aggregates showed a slight to moderate cytotoxic effect (relative cell viability of 85% to 60%) when applied at CPT concentrations between 10^−4^ and 10^−3^ mg/mL.

The half-maximal inhibitory concentration (IC_50_) values of CPT-loaded SF aggregates against MCF-7 cells, adjusted to CPT concentration, were determined (0.45 ± 0.07 µg/mL (CPT–SF1) > 0.20 ± 0.03 µg/mL (CPT–SF3) > (0.12 ± 0.04)^ns^ µg/mL (CPT) ~ (0.085 ± 0.015)^ns^ µg/mL (CPT–SF2).

To determine the effect of CPT-loaded SF aggregates on cell cycle distribution and cell death induction, Annexin V and PI staining were conducted after 48 h of treatment. CPT-loaded aggregates induced cell cycle arrest in the G2/M phase at a concentration of 0.27 mg/mL. In addition, this concentration resulted in significant cell death. CPT–SF nanoaggregates showed a superior cytotoxic effect on MCF-7 cells when compared to parent CPT, with about 30% to 56% of cell death in CPT-loaded SF aggregates and ~20% in parent CPT (Table S5). Interestingly, parent CPT provoked cell cycle arrest in the G2/M phase but did not induce cell death at the given concentration (Table 3 and Table S5).

**Figure 6:**
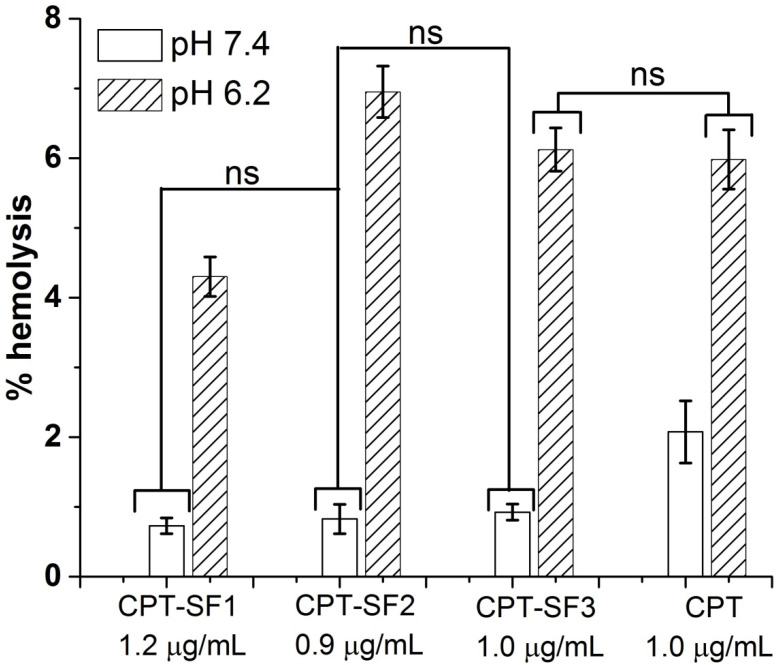
Percentage of hemolysis of RBC treated with CPT-loaded SF nanoaggregates and parent CPT in PBS at pH 7.4 and 6.2 (see structures in Figure 1). Mean ± standard deviation (*n* = 3). ns represent means with no significant differences (*p* > 0.05), but significant differences exist between non-marked bars (*p* < 0.05).

Figure 6 shows the hemolytic activity (% hemolysis) of CPT-loaded SF nanoaggregates and parent CPT on human red blood cells (RBC) at approximately 1 µg/mL of CPT in PBS (pH 6.2 and 7.4).

No hemolytic activity was observed for all CPT-loaded SF nanoaggregates (~1 µg/mL of CPT) at pH 7.4 (percentage of hemolysis below 1%) at 24 h. Parent CPT showed approximately 2% of hemolysis at pH 7.4. However, CPT–SF2, CPT–SF3 and parent CPT exhibited similar hemolytic activity at pH 6.2 (percentage of hemolysis approximately 6%). CPT–SF1 was slightly less hemolytic at this pH (percentage of hemolysis approximately 4%). Meanwhile, 0.1 mg/mL of blank SF nanoparticles had no hemolytic effect on RBCs at pH 7.4 and 6.2 (percentage of hemolysis below 1%) (Figure S15), while 1 µg/mL of CPT exerted significant hemolysis at pH 7.4 and 6.2 (~6%).

For that reason, SF aggregates can be considered as good candidates for the in vivo delivery of CPT. CPT-loaded SF aggregates might be directly injected into the tumor. Alternatively, CPT carrying SF aggregates could selectively accumulate in the cancer tissues via passive diffusion and the EPR effect [41,42]. Particularly, CPT–SF2 and CPT–SF3 soft aggregates might exhibit extended serum residence times and moderate reticuloendothelial system (RES) clearance [43]. Once CPT–SF2 or CPT–SF3 aggregates are inside the cancer cells or in the slightly acidic cancer tissue, the CPT cargo might be delivered faster because of enzyme-promoted and acidic hydrolysis degradation of SF–tocopherol and SF–testosterone carriers. Additional in vivo assessment of the bioavailability, biodistribution, antitumor activity, toxicity, and pharmacokinetics of the CPT-loaded SF aggregates must be carried out before their medical application.

**Figure 7:**
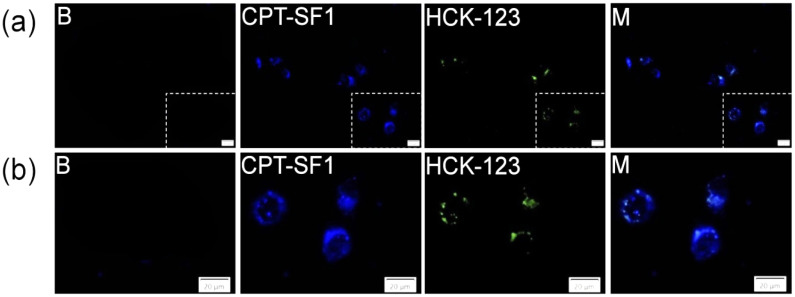
(**a**) MCF-7 cells fluorescence images and amplified area (**b**) of cells with LysoTracker (B), cells with 0.1 mg/mL of CPT–SF1, 50 nM of LysoTracker Yellow HCK-123 and combined pictures (M), scale bars are 20 µm (see structures in Figure 1).

To evaluate the uptake and localization of the CPT-loaded SF aggregates on MCF-7 cancer cells, fluorescence microscopy analyses were performed. Indeed, CPT–SF1 (Figure 7), CPT–SF2 and CPT–SF3 (Figures S16 and S17) internalized in cells (CPT associated with blue fluorescence) and accumulated in the lysosomes (LysoTracker HCK-123 associated with green fluorescence) [44]. The lysosomal accumulation of the CPT-loaded SF aggregates might facilitate the acidic hydrolysis of the vitamin–tyrosine and testosterone–tyrosine ester bonds in the SF aggregates. Thus, the co-delivery of vitamins, testosterone, and CPT inside the cancer cells is expected due to SF aggregates disintegration in the cancer cell lysosomes.

### 3.5. Spheroid Uptake and Cytotoxic Activity

**Figure 8:**
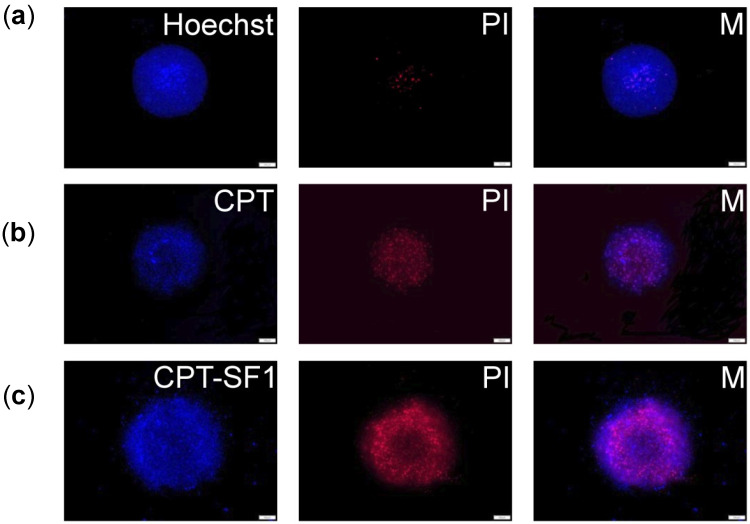
(**a**) MCF-7 spheroid fluorescence images without particles and PI or Hoechst staining. (**b**) Spheroids with 0.1 mg/mL of CPT, PI, and combined pictures (M); (**c**) Spheroids with 0.1 mg/mL of CPT–SF1, PI, and combined pictures (M). Scale bars are 100 µm (see structures in Figure 1).

MCF-7 cells were grown in 3D-like architecture as spheroids to assess the uptake and cytotoxicity of synthesized CPT-loaded SF nanoaggregates as a step forward to their intended medical use against human breast cancer. Fluorescence microscopy images (Figure 8 and Figure S18) show a good uptake of CPT-loaded SF nanoaggregates in MCF-7 spheroids (CPT associated blue fluorescence). It caused an extensive cell dead (PI related red fluorescence) after 72 h.

Cell death in MCF-7 spheroids treated with the CPT-loaded SF nanoaggregates was observed as a change of the spheroids borderline after 72 h when compared with no significant shape change of control (Figures S19 and S20).

The cell viability of MCF-7 spheroids was assessed after 72 h of treatment with CPT-loaded SF aggregates or parent CPT. The IC_50_ of CPT-loaded SF aggregates to MCF-7 spheroids, adjusted to anticancer drug concentration, showed significantly reduced cell viability for all nanoaggregates compared to parent CPT (1.75 ± 0.75 µg/mL (CPT) > 0.28 ± 0.03 µg/mL (CPT–SF2) > (0.14 ± 0.02)^ns^ µg/mL (CPT–SF3) ~ (0.13 ± 0.03)^ns^ µg/mL (CPT–SF1).

## 4. Conclusions

In this research, ergocalciferol-, tocopherol-, and testosterone-grafted SFs were prepared via the esterification of tyrosine fragments on SF, and CPT was efficiently encapsulated for its sustained delivery. The vitamins and testosterone grafting of SF attained a DS between 0.4 and 3.8 mol %, as determined by elemental analysis and ^1^H NMR spectroscopies. FTIR spectra and DSC thermograms showed that tocopherol and testosterone-grafted SF conjugates predominantly adopted a β-sheet conformation. The functionalization of SF with the vitamins and testosterone resulted in the almost doubled hydrodynamic sizes and zeta potentials of vitamins- and testosterone-grafted SF aggregates as compared to parent SF. TEM images showed 50–800 nm rounded and irregular flake-like SF aggregates, which is in good agreement with DLS results. The dialysis–precipitation method allowed efficient CPT encapsulation in the SF aggregates, with a CPT content of 6.3–8.3 wt %. A significant shrinkage of SF aggregates in PBS was observed once CPT was encapsulated in the hydrophobic core. Controlled and almost quantitative CPT release was achieved after 6 days in PBS at 37 °C, with almost linear release during the first 8 h. The antiproliferative effect of CPT on MCF-7 cancer cells seems unaltered after encapsulation in the SF aggregates. Particularly, CPT-loaded tocopherol-grafted SF aggregates showed slightly more cytotoxicity against MCF-7 cells than parent CPT. CPT-loaded SF aggregates provoked significantly reduced MCF-7 cell viability, with much lower IC_50_ values than parent CPT in MCF-7 spheroids. It might indicate a better uptake of the loaded SF aggregates. All CPT-loaded SF aggregates caused significant cell cycle arrest in the G2/M phase with significant cell death, whereas parent CPT provoked significant cell cycle arrest in the G2/M phase and slight necrosis. No hemolytic activity was observed for the blank SF aggregates at pH 7.4 and 6.2. CPT-loaded SF aggregates provoked no hemolysis at pH 7.4 and caused similar or slightly lower hemolysis of human RBC than parent CPT at pH 6.2. Fluorescence microscopy imaging confirmed MCF-7 cells uptake and lysosomal accumulation of CPT-loaded SF aggregates as well as good cellular uptake in the MCF-7 spheroids. Accordingly, CPT-loaded SF aggregates are good candidates for antitumor therapy. Future studies may focus on the in vivo evaluation of antitumor activity, toxicity, biodistribution, and other pharmacokinetic parameters.

## Figures and Tables

**Table 1:** Degree of substitution (DS %) and yield (Y %) of synthesized SF derivatives. Number average molecular weight (M_n_) of raw SF.

Sample	C/N ^a^	DS ^b^ (%)	DS ^c^ (%)	Y (%)	M_n_^d^ (kg/mol)	M_w_/M_n_^d^
SF	2.6049	-		-	27.2	1.22
SF1	3.6218	3.2	3.0	71	-	-
SF2	3.8355	3.8	3.6	64	-	-
SF3	2.7061	0.4	-	58	-	-

^a^ Carbon to nitrogen ratio measured by elemental analysis. ^b^ DS estimated from the carbon to nitrogen ratio. ^c^ DS calculated from the ^1^H NMR. ^d^ M_n_ and polydispersity index estimated using GPC.

**Table 2:** Hydrodynamic parameters of SF aggregates; AFM average diameters of raw and modified SF dried particles; CPT contents in weight (wt %) and CPT encapsulation efficiency (EE %) of CPT-loaded SF aggregates.

Sample	dh ^a^ (nm)/(PDI)	dh ^b^ (nm)/(PDI)	ζ ^a^ (mV)	d_AFM_ (nm)	wt %	EE %
SF	216 ± 7 (0.5)	260 ± 4 (0.45)	−14.8 ± 0.8	-	-	-
SF1	554 ± 3 (0.32)	471 ± 7 (0.37)	−32.2 ± 0.4	75 ± 10	-	-
SF2	653 ± 1 (0.45)	727 ± 4 (0.15)	−28.3 ± 0.5	53 ± 9 ^ns^	-	-
SF3	620 ± 3 (0.31)	824 ± 6 (0.28)	−30.8 ± 0.3	45 ± 7 ^ns^	-	-
CPT–SF1	-	420 ± 5 (0.5)	-	-	8.3	84
CPT–SF2	-	370 ± 7 (0.51)	-	-	6.3	60
CPT–SF3	-	340 ± 5 (0.34)	-	-	6.8	67

^a^ Samples in H_2_O. ^b^ Samples in PBS. ns represent means with no significant differences (*p* > 0.05).

**Table 3:** MCF-7 cell cycle arrest parameters at control and 0.27 mg/mL of CPT-loaded SF nanoaggregates and parent CPT.

Sample	G0, G1 (%)	S (%)	G2, M (%)
Control	69 ± 7	7 ± 1	23 ± 6
CPT–SF1	54.4 ± 0.5	7.3 ± 0.6	38.6 ± 0.7
CPT–SF2	38 ± 2	3 ± 2	51 ± 13
CPT–SF3	60 ± 3	5.51 ± 0.07	34 ± 3
CPT	40 ± 2	4 ± 2	55 ± 1

## Data Availability

Data available in the Supplementary Material.

## Supplementary Materials

The following are available online at https://www.mdpi.com/article/10.3390/polym13213804/s1, Figure S1: UV spectra of CPT at 0.00503 mg/mL, CPT–SF1 at 0.025 mg/mL, CPT–SF2 at 0.0234 mg/mL, CPT–SF3 at 0.0253 mg/mL in DMSO, Figure S2: Calibration curve of CPT in DMSO, Figure S3: Calibration curve of CPT in PBS (pH 7.4), Figure S4: IR spectra of ergocalciferol hemisuccinate (MSVitD2), tocopherol hemisuccinate (MSToc) and testosterone hemisuccinate (MSTest), Figure S5: TGA curve of SF2, Figure S6: TGA curve of SF3, Figure S7: TGA curve of parent SF, Figure S8: ^1^H NMR spectrum of parent SF in DMSO-d_6_, Figure S9: ^1^H NMR spectrum of SF2 in DMSO-d_6_, Figure S10: ^1^H NMR spectrum of SF3 in DMSO-d_6_, Figure S11: TEM micrographs of SF1–SF3 dried aggregates at 21,000× magnification, Figure S12: Linear fitting of log(Cumulative Release (%)) vs. log(Time(hours)) of CPT-loaded SF aggregates up to 120 h in PBS (pH 7.4) at 37 °C, Figure S13: ATR-FTIR spectra of CPT and CPT-loaded SF aggregates, Figure S14: Relative viability of MCF-7 cells with parent SF at 0.2 mg/mL and blank SF1–SF3 aggregates at 0.1 mg/mL. Mean ± standard deviation (*n* = 3). ns represent means with no significant differences (*p* > 0.05), Figure S15: Percentage of hemolysis of RBC treated with 0.1 mg/mL of blank SF nanoaggregates in PBS at pH 7.4 and 6.2 (see structures in Figure 1). Mean ± standard deviation (*n* = 3). * groups of means with significant differences (*p* < 0.05), Figure S16: (a) MCF-7 cells confocal images and amplified areas (b) of cells without particles and LysoTracker (B), cells with 0.1 mg/mL of CPT–SF2 aggregates, 50 nM of LysoTracker Yellow HCK-123, and combined pictures (M). Scale bars are 20 µm (see structures in Figure 1), Figure S17: (a) MCF-7 cells confocal images and amplified areas (b) of cells without particles and LysoTracker (B), cells with 0.1 mg/mL of CPT–SF3 aggregates, 50 nM of LysoTracker Yellow HCK-123, and combined pictures (M). Scale bars are 20 µm (see structures in Figure 1), Figure S18: (a) MCF-7 spheroid fluorescence images with 0.1 mg/mL of CPT–SF2 aggregates, PI, and combined pictures (M). (b) Spheroids with 0.1 mg/mL of CPT-SF3 aggregates, PI, and combined pictures (M). Scale bars are 100 µm (see structures in Figure 1), Figure S19: MCF-7 spheroid bright field images without particles (B) or with 0.1 mg/mL of CPT or CPT–SF1 nanoaggregates at: 0 h (a) and 72 h (b). Scale bars are 100 µm (see structures in Figure 1), Figure S20: MCF-7 spheroid bright field images with 0.1 mg/mL of CPT–SF2 or CPT–SF3 nanoaggregates at: 0 h (a) and 72 h (b). Scale bars are 100 µm (see structures in Figure 1), Table S1: AFM average diameters and areas of dried SF aggregates, Table S2: Linear fitting parameters of *in vitro* CPT release profiles of SF aggregates up to 8 h (intercept 0, slope k, adjusted R-Square) in PBS (pH 7.4) at 37 °C, Table S3: SWeibull2 fitting parameters of Cumulative Release(%) vs. Time(hours) of CPT-loaded SF aggregates up to 120 h (Cumulative Release (%) = a − (a − b)*exp(−(k*Time(hours))^d^) in PBS (pH 7.4) at 37 °C, Table S4: Linear fitting parameters of log(Cumulative Release(%)) vs. log(Time(hours)) of CPT-loaded SF conjugates up to 120 h (intercept m, slope k, adjusted R-Square) in PBS (pH 7.4) at 37 °C, and wavenumber of CPT C=O (υ(C=O)) IR adsorption, Table S5: MCF-7 cell cytotoxicity at control and 0.27 mg/mL of CPT-loaded SF nanoaggregates, and parent CPT determined by Annexin V and PI, Characterization data for parent SF and SF1–SF3 conjugates.
