# Supplementary material for: Self-Assembled Silk Fibroin-Based Aggregates for Delivery of Camptothecin
Source: Polymers (Basel). 2021 Nov 3;13(21):3804. doi: 10.3390/polym13213804 (PMC8587969; doi:10.3390/polym13213804)
Supplement: Supplementary file 1 [file polymers-13-03804-s001.zip › polymers-1426937-sup.pdf]

polymers

MDPI

Article

Self-Assembled Silk Fibroin-Based Aggregates for Delivery of Camptothecin

Javier Pérez Quiñones [1,*,†], Cornelia Roschger [2,†], Andreas Zierer [2], Carlos Peniche-Covas [3] and Oliver Brüggemann [1]

[1] Institute of Polymer Chemistry, Johannes Kepler University Linz, Altenberger Straße 69, 4040 Linz, Austria; oliver.brueggemann@jku.at (O.B.)
[2] Department for Cardiac-, Vascular- and Thoracic Surgery, Johannes Kepler University Linz, Kepler University Hospital GmbH, Altenberger Straße 69, 4040 Linz and Krankenhausstraße 7a, 4020 Linz, Austria; cornelia.roschger@jku.at (C.R.); andreas_florian.zierer@jku.at or andreas.zierer@kepleruniklinikum.at (A.Z.)
[3] Facultad de Química, Universidad de La Habana, Zapata S/N entre G y Carlitos Aguirre, La Habana 10400, Cuba; cpeniche2015@yahoo.com (C.P.-C.)
* Correspondence: javenator@gmail.com or javier.perez_quinones@jku.at (J.P.Q.); Tel.: +43-670-4039820
† These authors contributed equally.

1. Supporting Tables

Table S1. AFM average diameters and areas of dried SF aggregates.

Samples	Counts	Average diameter (nm)	Average area (nm^2)
SF1	27	75 ± 10	5567 ± 1588
AFM_SF1NotInPaper.jpg	30	66.1	4640
AFM_SF1NotReported.jpg	24	85.6	7400
AFM_SF1_0004.jpg	27	72.6	4660
SF2	46	53 ± 9	2780 ± 867
AFM_SF2_0001.jpg	47	62.4	3730
AFM_SF2NotInPaper_0003.jpg	56	48.4	2580
AFM_SF2NotReported.jpg	35	46.8	2030
SF3	130	45 ± 7	1463 ± 408
AFM_SF3_0000.jpg	148	37.2	1010
AFM_SF3NotInPaper_0001.jpg	117	48.2	1580
AFM_SF3NotReported.jpg	124	50.5	1800

Table S2. Linear fitting parameters of *in vitro* CPT release profiles of SF aggregates up to 8 h (intercept 0, slope k, adjusted R-Square) in PBS (pH 7.4) at 37 °C.

Samples	k	Adjusted R-Square
CPT–SF1	1.23 ± 0.04	0.9950
CPT–SF2	2.44 ± 0.02	0.9997
CPT–SF3	1.90 ± 0.02	0.9994

Table S3. SWeibull2 fitting parameters of Cumulative Release(%) *vs.* Time(hours) of CPT-loaded SF aggregates up to 120 h (Cumulative Release (%) = a – (a – b)*exp(–(k*Time(hours))^d) in PBS (pH 7.4) at 37 °C.

Samples	a	b	k	d	Adjusted R-Square
CPT–SF1	124 ± 58	3 ± 2	0.007 ± 0.005	1.1 ± 0.2	0.9918
CPT–SF2	116 ± 25	6 ± 6	0.014 ± 0.005	1.1 ± 0.4	0.9760
CPT–SF3	138 ± 69	7 ± 4	0.008 ± 0.006	1.2 ± 0.4	0.9812

Table S4. Linear fitting parameters of log(Cumulative Release(%)) *vs.* log(Time(hours)) of CPT-loaded SF conjugates up to 120 h (intercept m, slope k, adjusted R-Square) in PBS (pH 7.4) at 37 °C, and wavenumber of CPT C=O (υ(C=O)) IR adsorption.

Samples	m	k	R^2	υ(C=O)
CPT–SF1	0.23 ± 0.03	0.78 ± 0.02	0.9939	1732
CPT–SF2	0.56 ± 0.05	0.72 ± 0.04	0.9825	1739
CPT–SF3	0.42 ± 0.05	0.75 ± 0.03	0.9837	1739

Table S5. MCF-7 cell cytotoxicity at control and 0.27 mg/mL of CPT-loaded SF nanoaggregates, and parent CPT determined by Annexin V and PI.

Sample	Alive (%)	Necrosis (%)	Early apoptosis (%)	Late apoptosis (%)
Control	95 ± 3	1.9 ± 0.6	2 ± 2	1.1 ± 0.6
CPT–SF1	44 ± 2	9 ± 9	25 ± 13	23 ± 3
CPT–SF2	70 ± 10	9 ± 6	9 ± 14	18 ± 15
CPT–SF3	56 ± 5	11 ± 4	15 ± 5	20 ± 3
CPT	80 ± 7	13 ± 13	4 ± 5	3 ± 2

2. Characterization data for parent SF and SF1–SF3 conjugates

SF: ATR-FTIR (solid) *υ* max: 3291 (N–H), 3070 (C–H aromatic), 2937 (C–H aliphatic), 1645 (Amide I, silk I), 1535 (Silk I), 1515 (Silk II), 1453 (C–H bend), 1234 (C–N), 1169 (C–H in-plane bending); ^{1}H NMR (300 MHz, 298 K, $CDCl_3$): δ = 0.84 (s, 1H, CH_3- of valine), 6.7-8.2 (m, 4.32H, aromatic protons of tyrosine and phenylalanine). GPC (g/mol) M_n = 15048, Mw = 22121. Elemental analysis (experimental): %C 44.83, %H 6.31, %N 17.21

SF1: ATR-FTIR (solid) *υ* max: 3284 (N–H), 3072 (C–H aromatic), 2937 (C–H aliphatic), 1732 (C=O, ergocalciferol-succinate bond + ergocalciferol succinate-Tyrosine of SF bond), 1643 (Amide I, silk I), 1532 (Silk I), 1518 (Silk II), 1453 (C–H bend), 1240 (C–N), 1162 (C–H in-plane bending + C–O–C); ^{1}H NMR (300 MHz, $CDCl_3$): δ = 0.82-1.02 (d, 9.37H, CH_3- of valine + H18 + H21 + H26 + H27 + H28), 5.27-5.35 (d, 0.82H, H6 + H7), 6.7-8.2 (m, 3.31H, aromatic protons of tyrosine and phenylalanine). Elemental analysis (experimental): %C 50.85, %H 6.60, %N 14.04

SF2: ATR-FTIR (solid) *υ* max: 3285 (N–H), 3075 (C–H aromatic), 2928 (C–H aliphatic), 1739 (C=O, tocopherol-succinate bond + tocopherol succinate-Tyrosine of SF bond), 1624 (Amide II, silk II), 1514 (Silk II), 1448 (C–H bend), 1229 (C–N), 1155 (C–H in-plane bending + C–O–C); ^{1}H NMR (300 MHz, $CDCl_3$): δ = 0.84 (s, 9.10H, CH_3- of valine + 4′CH_3- + 8′CH_3- + 12′CH_3-), 6.7-8.2 (m, 3.84H, aromatic protons of tyrosine and phenylalanine). Elemental analysis (experimental): %C 52.70, %H 7.06, %N 13.74

SF3: ATR-FTIR (solid) *υ* max: 3282 (N–H), 3073 (C–H aromatic), 2935 (C–H aliphatic), 1737 (C=O, testosterone-succinate bond + testosterone succinate-Tyrosine of SF bond), 1699 (C=O, SF-succinate bond), 1622 (Amide II, silk II), 1514 (Silk II), 1446 (C–H bend), 1230 (C–N), 1168 (C–H in-plane bending + C–O–C); ^{1}H NMR (300 MHz, $CDCl_3$): δ = 0.84-0.86 (m, 1.38H, CH_3- of valine + H18 + H19), 5.34 (s, 0.31, H4), 6.7-8.2 (m, 2.41H, aromatic protons of tyrosine and phenylalanine). Elemental analysis (experimental): %C 46.03, %H 6.28, %N 17.01

3. Supporting Figures

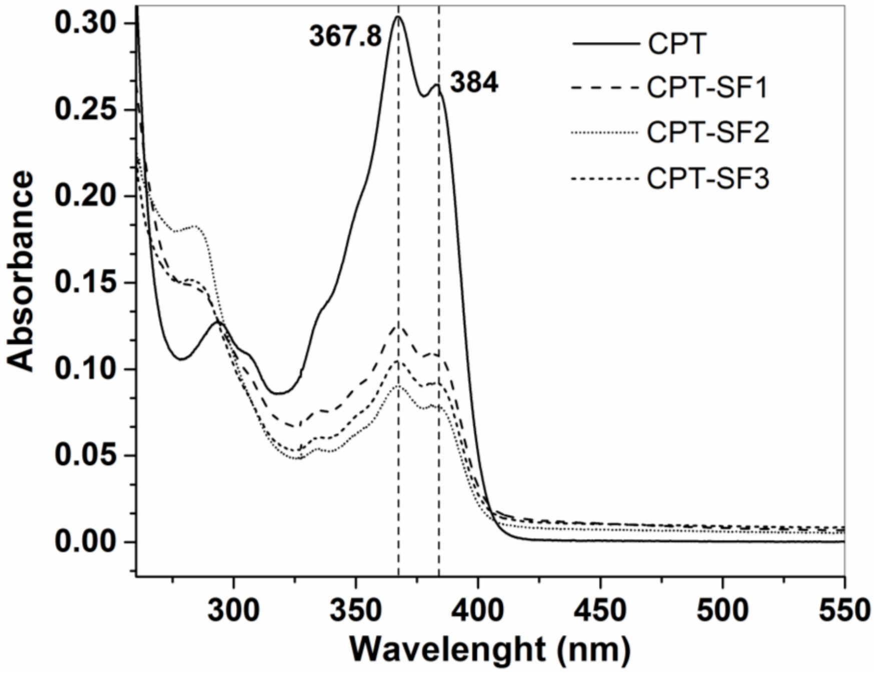

Figure S1. UV spectra of CPT at 0.00503 mg/mL, CPT–SF1 at 0.025 mg/mL, CPT–SF2 at 0.0234 mg/mL, CPT–SF3 at 0.0253 mg/mL in DMSO.

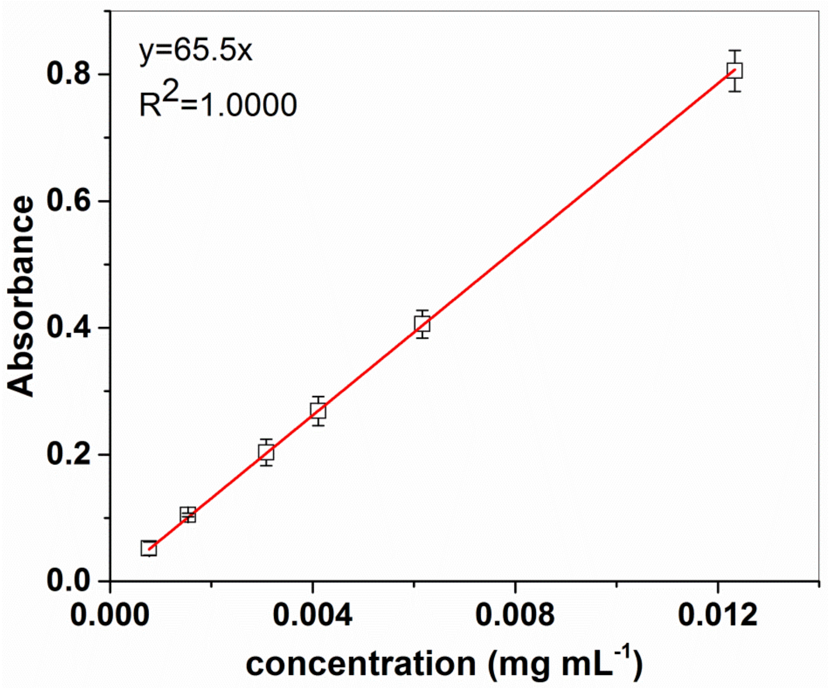

Figure S2. Calibration curve of CPT in DMSO.

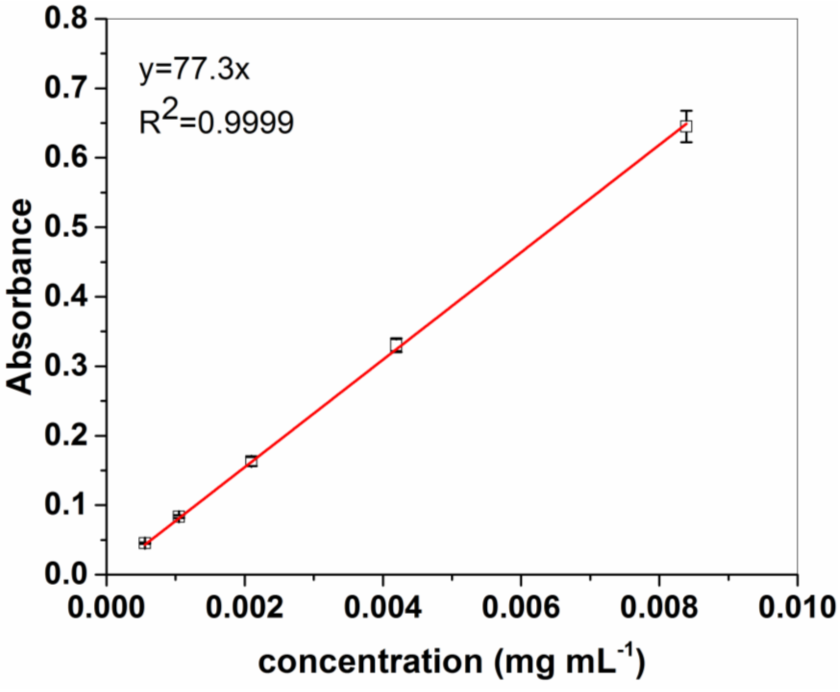

Figure S3. Calibration curve of CPT in PBS (pH 7.4).

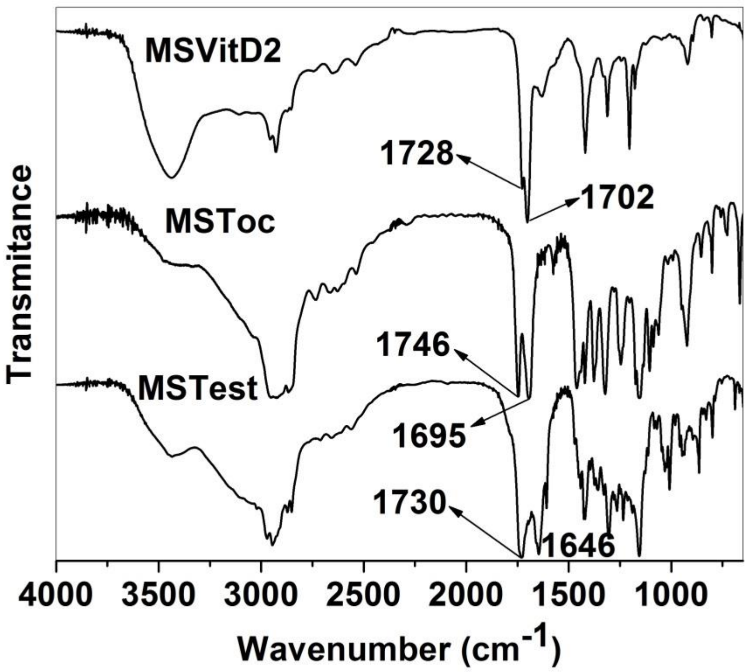

Figure S4. IR spectra of ergocalciferol hemisuccinate (MSVitD2), tocopherol hemisuccinate (MSToc) and testosterone hemisuccinate (MSTest).

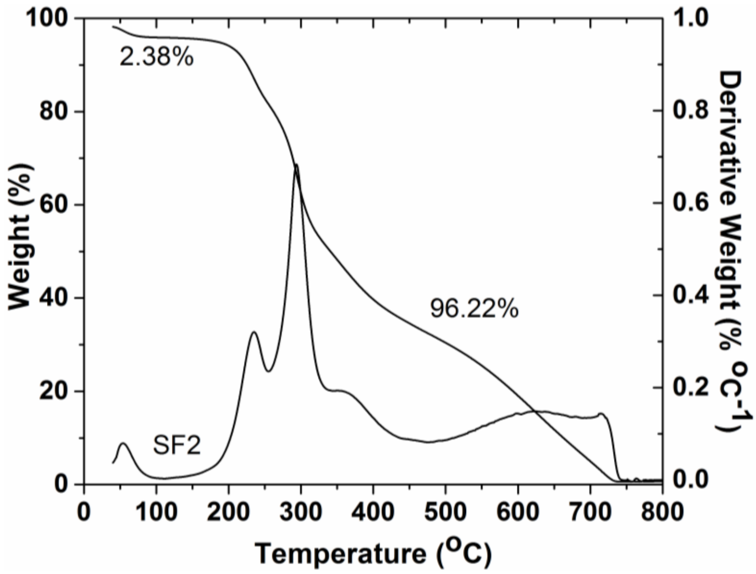

Figure S5. TGA curve of SF2.

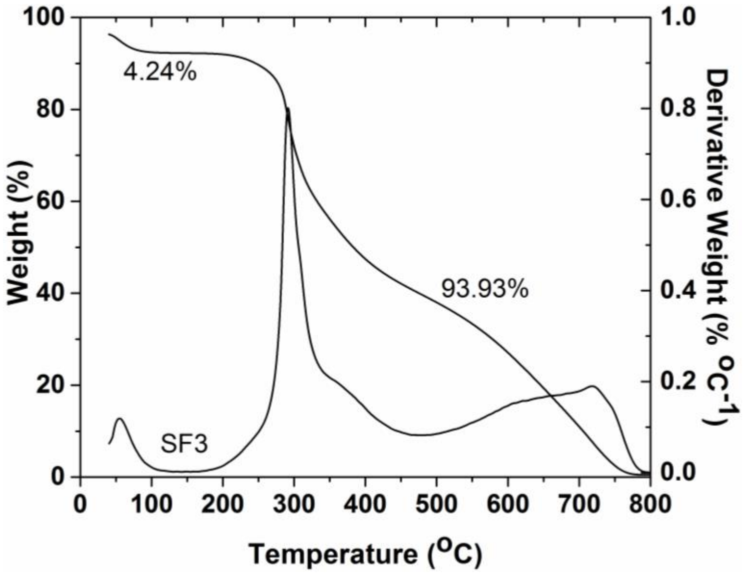

Figure S6. TGA curve of SF3.

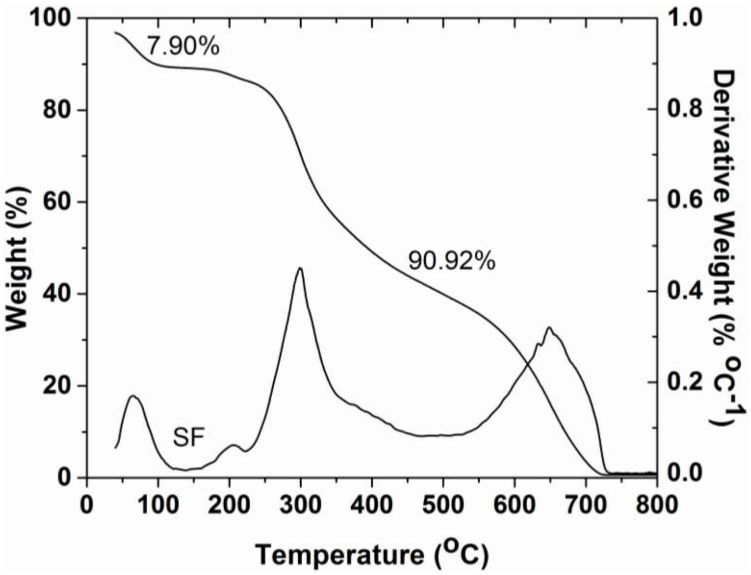

Figure S7. TGA curve of parent SF.

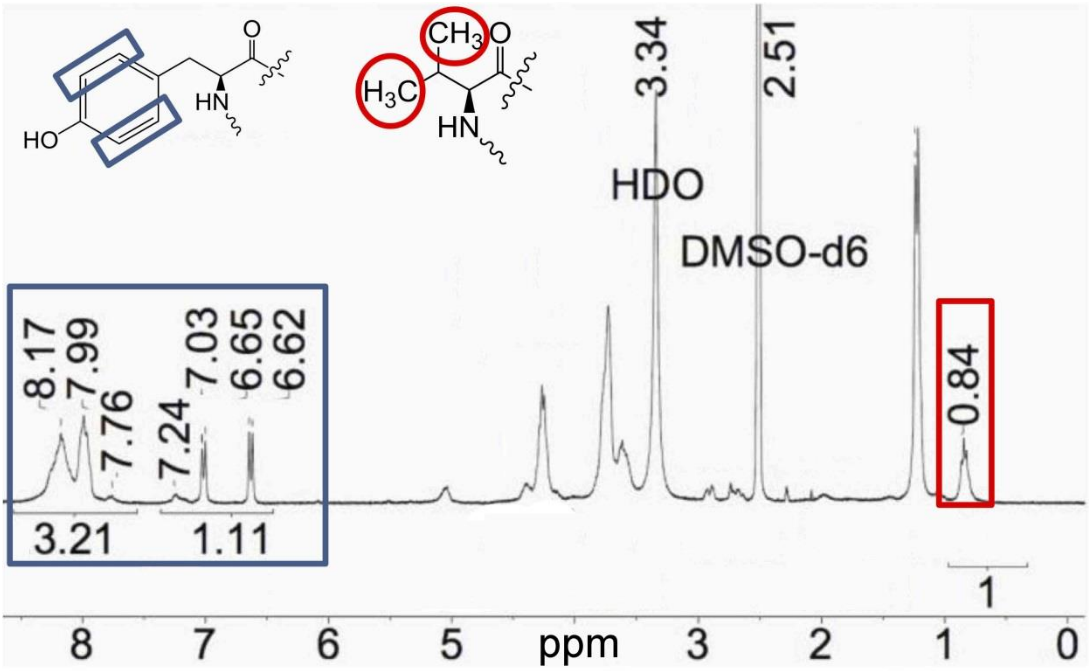

Figure S8. ^{1}H NMR spectrum of parent SF in DMSO-d_6.

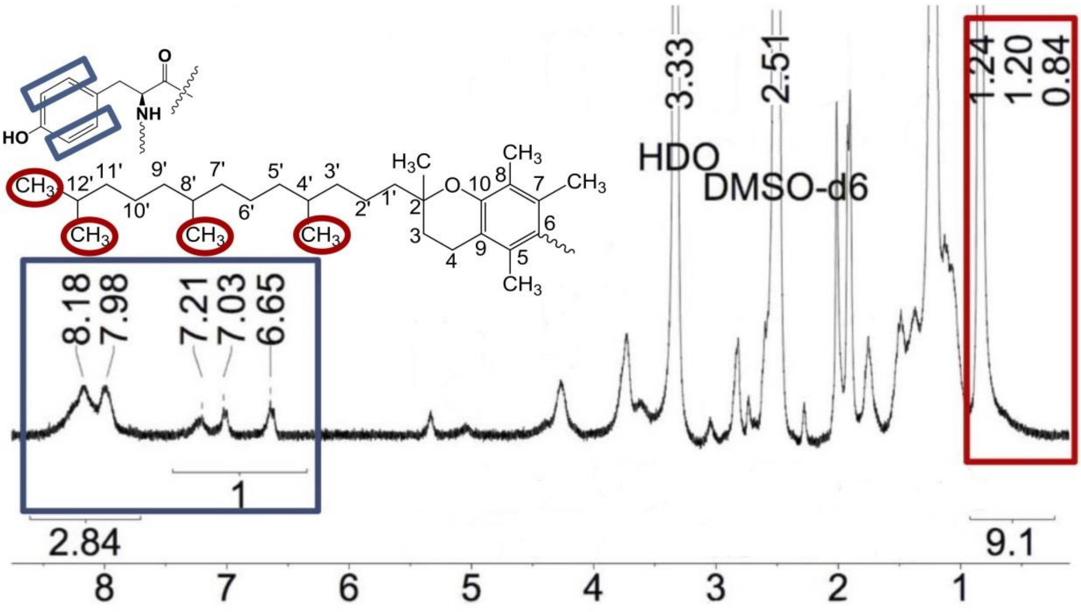

Figure S9. ^{1}H NMR spectrum of SF2 in DMSO-d_6.

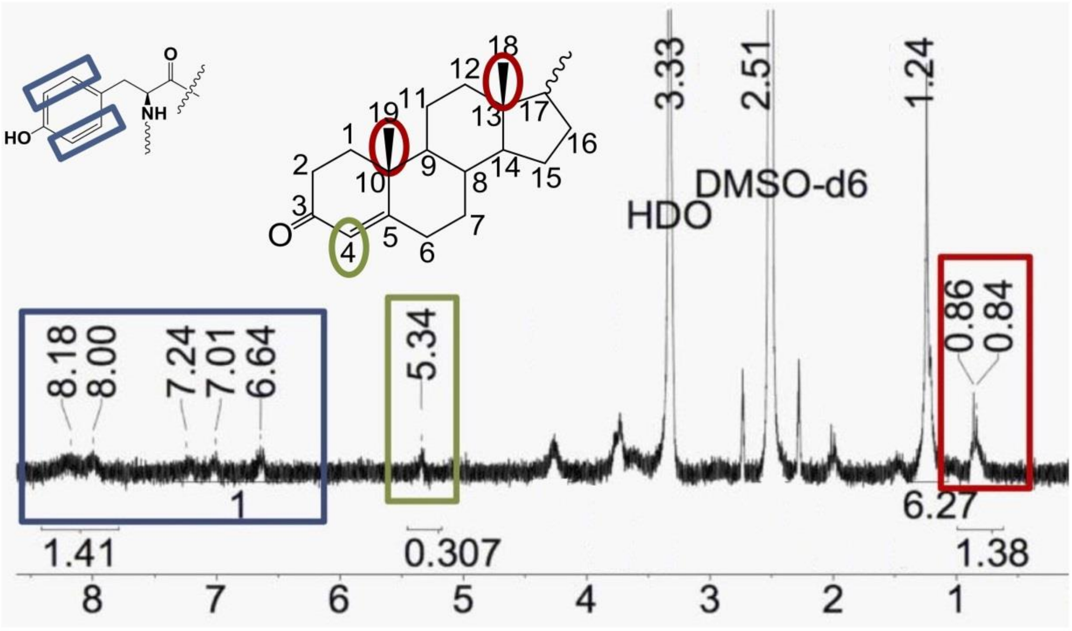

Figure S10. ^{1}H NMR spectrum of SF3 in DMSO-d_6.

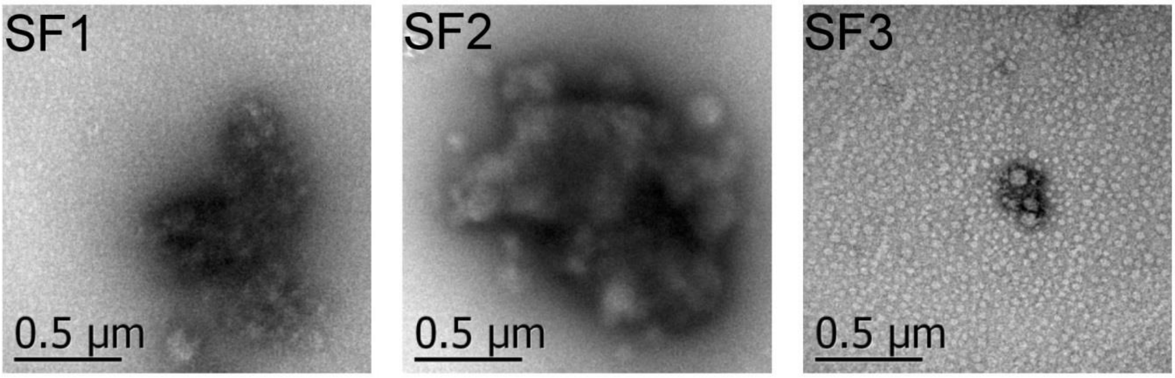

Figure S11. TEM micrographs of SF1–SF3 dried aggregates at 21,000× magnification.

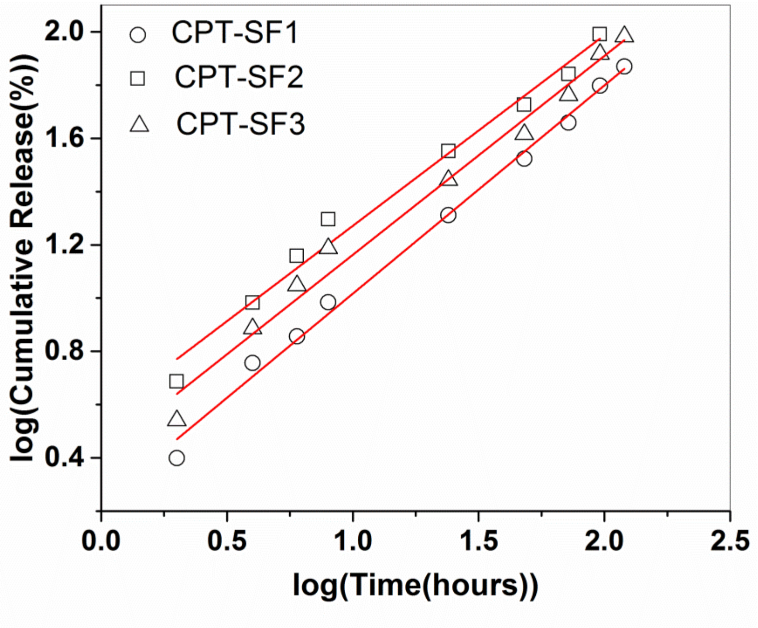

Figure S12. Linear fitting of log(Cumulative Release (%)) vs. log(Time(hours)) of CPT-loaded SF aggregates up to 120 h in PBS (pH 7.4) at 37 °C

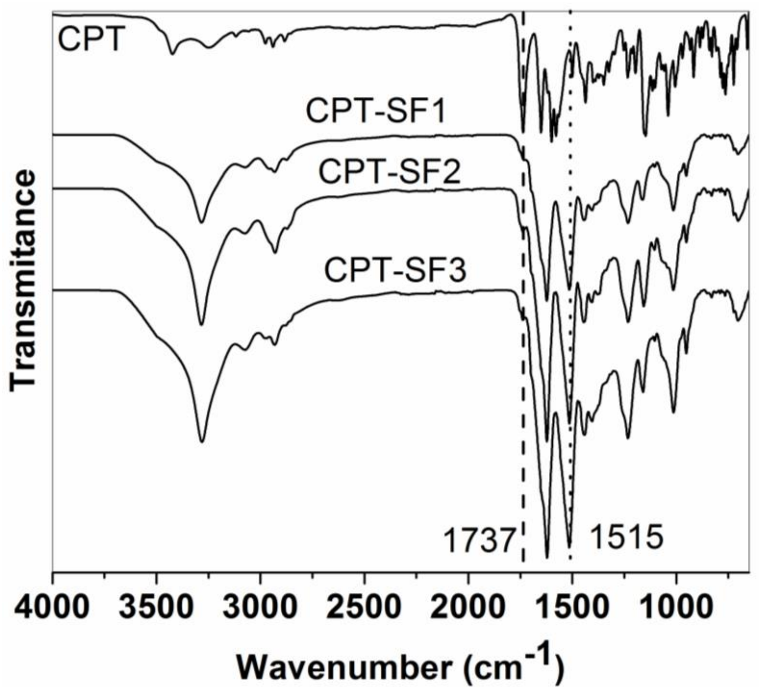

Figure S13. ATR-FTIR spectra of CPT and CPT-loaded SF aggregates.

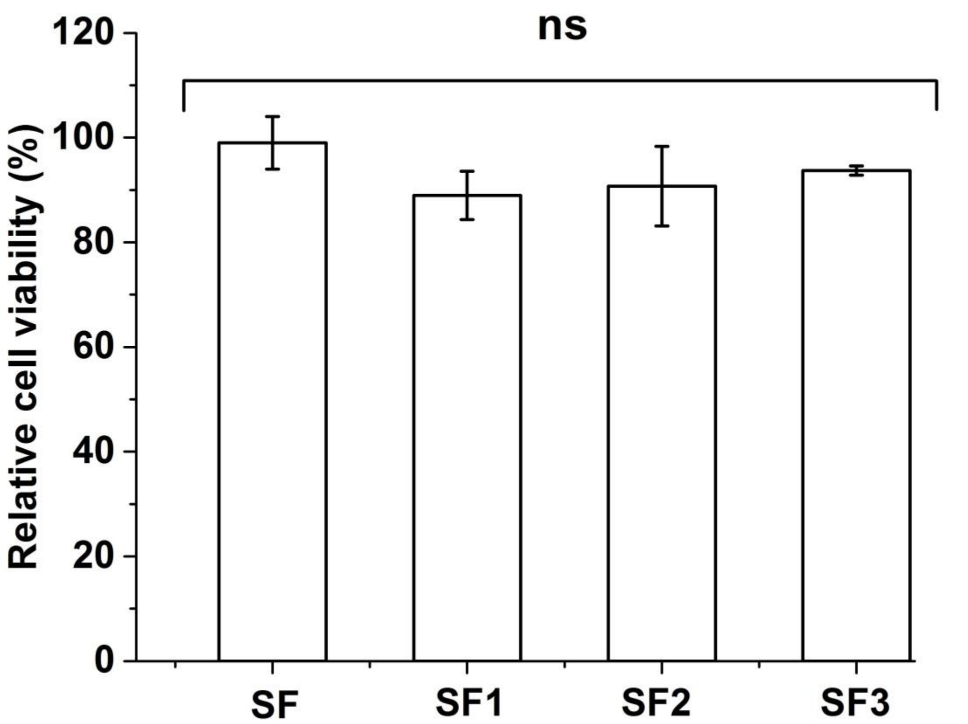

Figure S14. Relative viability of MCF-7 cells with parent SF at 0.2 mg/mL and blank SF1–SF3 aggregates at 0.1 mg/mL. Mean ± standard deviation (n = 3). ns represent means with no significant differences ($p > 0.05$).

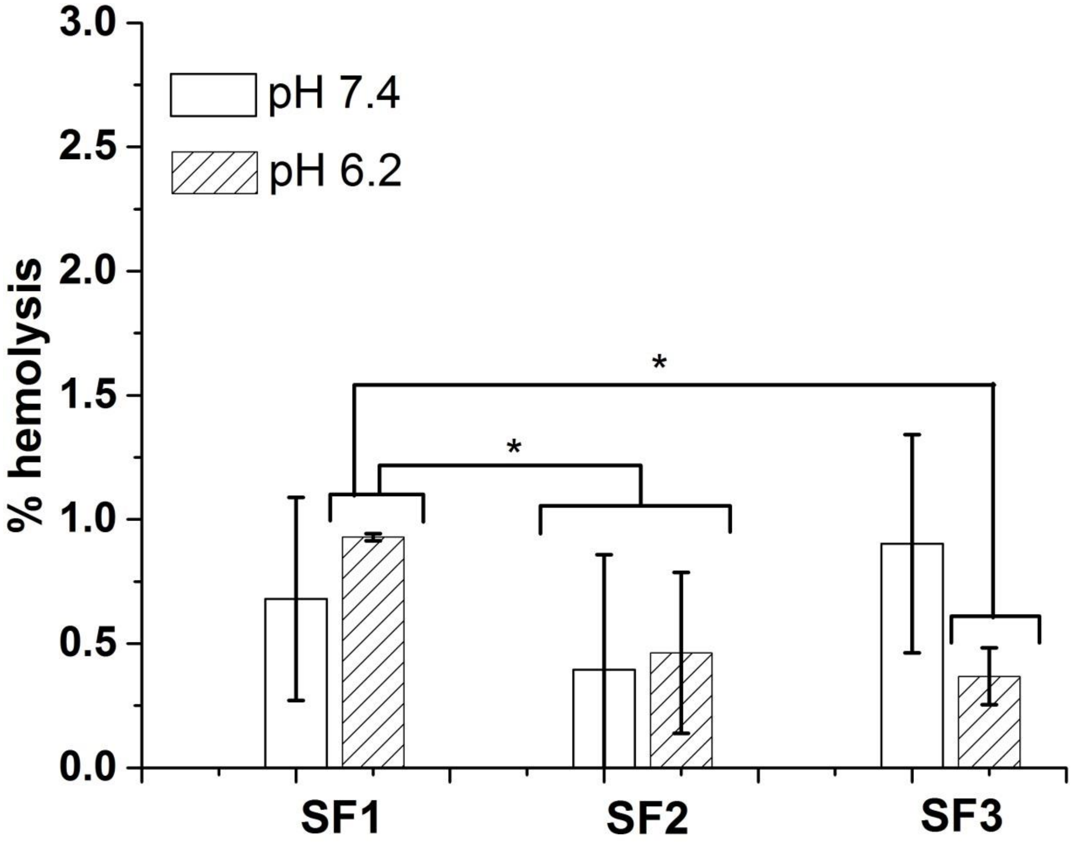

Figure S15. Percentage of hemolysis of RBC treated with 0.1 mg/mL of blank SF nanoaggregates in PBS at pH 7.4 and 6.2 (see structures in Figure 1). Mean ± standard deviation (n = 3). * groups of means with significant differences (p < 0.05).

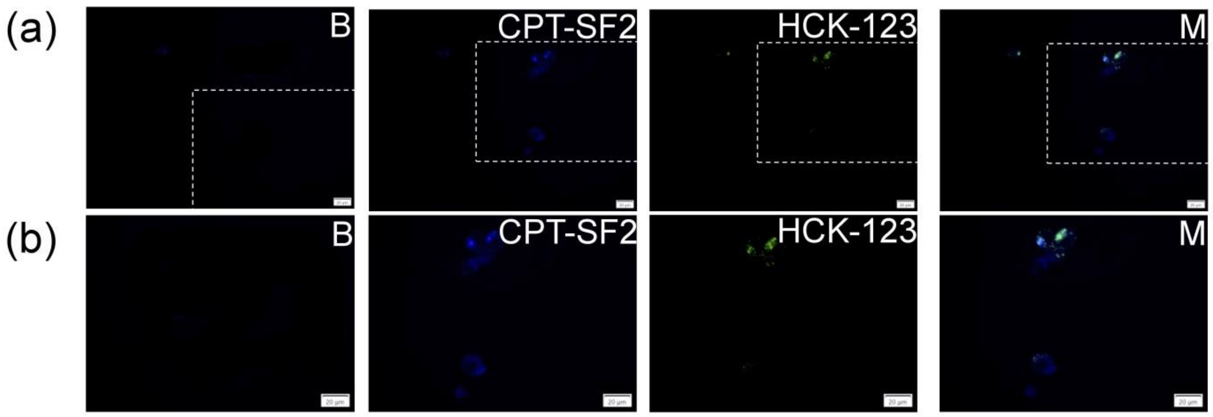

Figure S16. (a) MCF-7 cells confocal images and amplified areas (b) of cells without particles and LysoTracker (B), cells with 0.1 mg/mL of CPT–SF2 aggregates, 50 nM of LysoTracker Yellow HCK-123, and combined pictures (M). Scale bars are 20 μm (see structures in Figure 1).

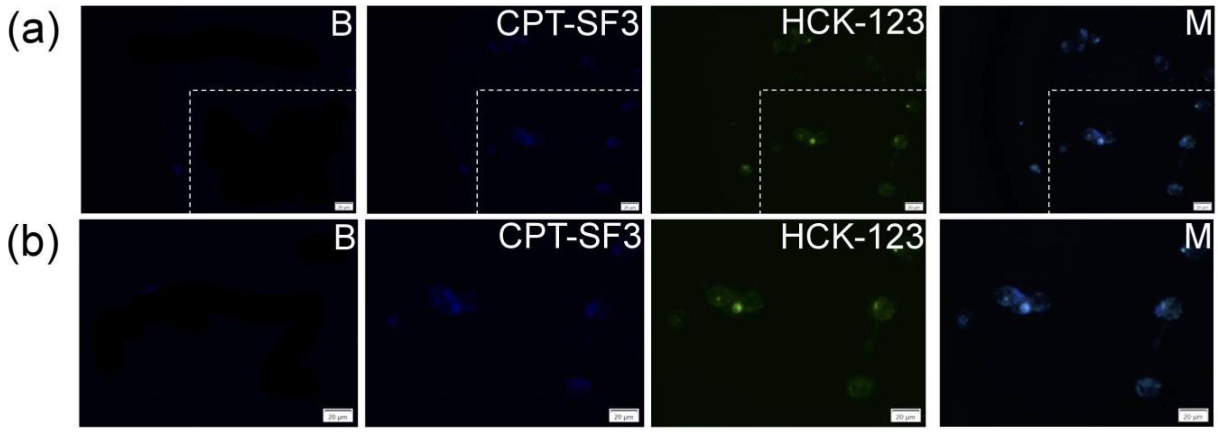

Figure S17. (a) MCF-7 cells confocal images and amplified areas (b) of cells without particles and LysoTracker (B), cells with 0.1 mg/mL of CPT–SF3 aggregates, 50 nM of LysoTracker Yellow HCK-123, and combined pictures (M). Scale bars are 20 μm (see structures in Figure 1).

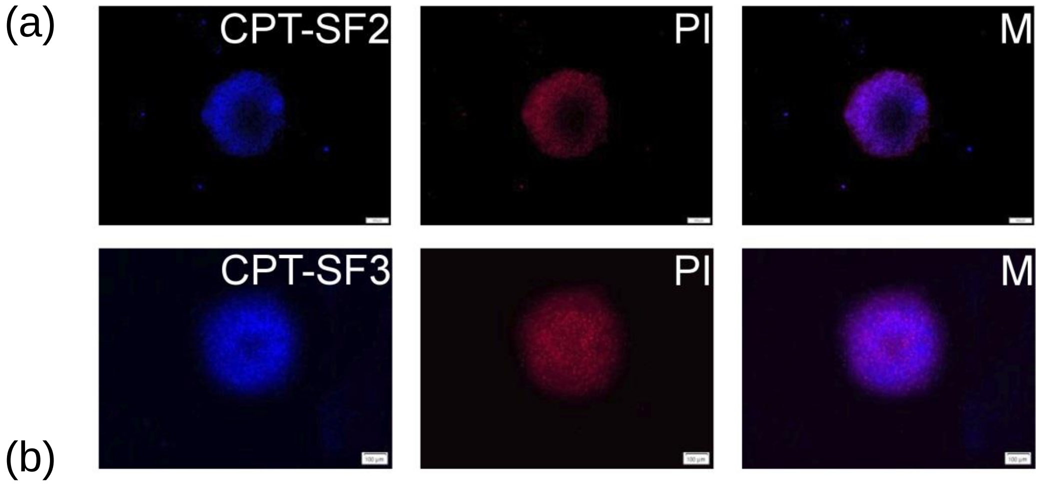

Figure S18. (a) MCF-7 spheroid fluorescence images with 0.1 mg/mL of CPT–SF2 aggregates, PI, and combined pictures (M). (b) Spheroids with 0.1 mg/mL of CPT–SF3 aggregates, PI, and combined pictures (M). Scale bars are 100 μm (see structures in Figure 1).

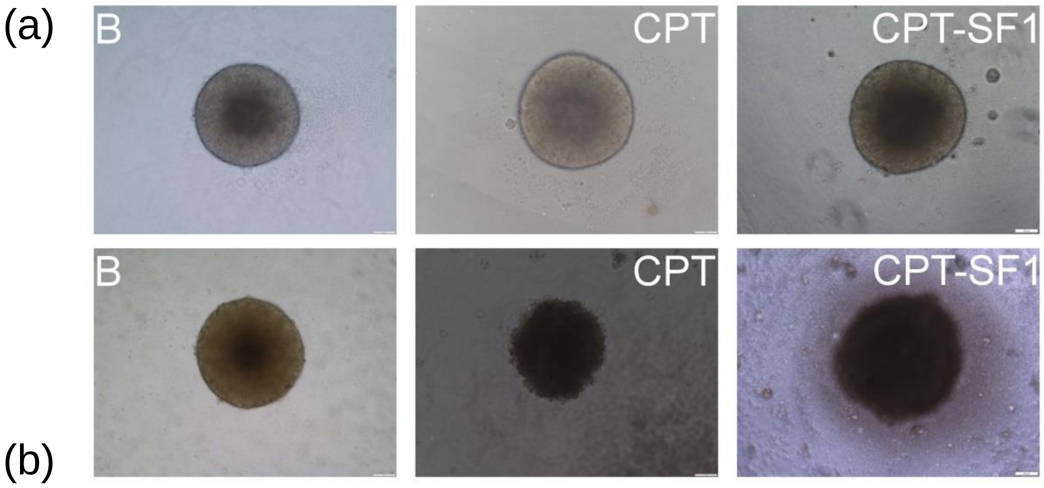

Figure S19. MCF-7 spheroid bright field images without particles (B) or with 0.1 mg/mL of CPT or CPT–SF1 nanoaggregates at: 0 h (a) and 72 h (b). Scale bars are 100 µm (see structures in Figure 1).

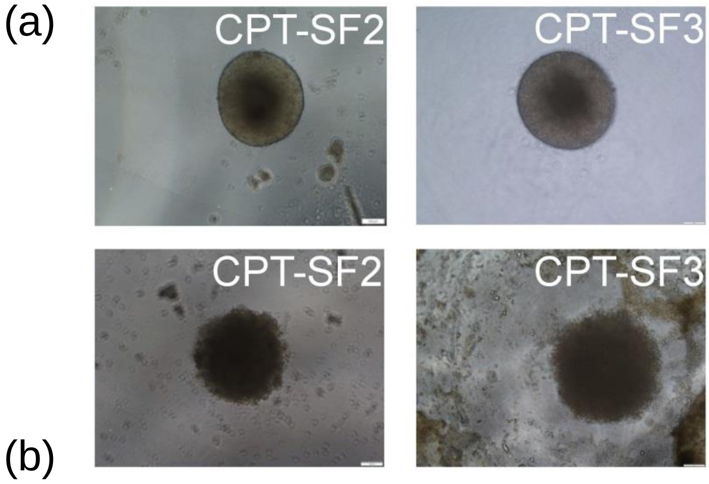

Figure S20. MCF-7 spheroid bright field images with 0.1 mg/mL of CPT-SF2 or CPT–SF3 nanoaggregates at: 0 h (a) and 72 h (b). Scale bars are 100 µm (see structures in Figure 1).
